# Efficient Microwave-Assisted Palladium-Catalyzed Selective *N*-Arylation of Anilines with 2,3-Dihalopyridines in Water

**DOI:** 10.3390/ma19051003

**Published:** 2026-03-05

**Authors:** Hao-Chun Hu, Cheng-Yi Chen, Shyh-Chyun Yang

**Affiliations:** 1 Department of Fragrance and Cosmetic Science, College of Pharmacy, Kaohsiung Medical University, Kaohsiung 807378, Taiwan; drjcount@livemail.tw; 2 School of Pharmacy, College of Pharmacy, Kaohsiung Medical University, Kaohsiung 807378, Taiwan; chengyi716@gmail.com; 3 Department of Medical Research, Kaohsiung Medical University Hospital, Kaohsiung 80756, Taiwan; 4 School of Pharmacy, College of Pharmacy, Taipei Medical University, Taipei 11031, Taiwan

**Keywords:** microwave, palladium catalysis, *N*-arylation, aminopyridines, green chemistry

## Abstract

Under aqueous conditions, transition-metal catalysis offers an attractive platform for greener C–N bond formation by reducing reliance on hazardous organic solvents. Herein, we report a microwave-assisted palladium-catalyzed selective *N*-arylation of anilines with 2,3-dihalopyridines in water. Systematic optimization revealed that a catalyst system comprising PdCl_2_(1,10-phenanthroline)_2_ and (±)-BINAP in the presence of K_3_PO_4_ enables efficient coupling under microwave irradiation. Under the optimized conditions (PdCl_2_(1,10-Phenanthroline)_2_, 2 mol%; (±)-BINAP, 3 mol%; K_3_PO_4_, 3.5 equiv; H_2_O, 2.5 mL; 150 °C; 30 min), the coupling of aniline with 2,3-dichloropyridine afforded the corresponding aminopyridine product in up to 91% isolated yield. The method was extended to various 2,3-dihalopyridines and substituted anilines, providing moderate to excellent yields with good regioselectivity. Mechanistically, the transformation is consistent with a Pd(0)/Pd(II) catalytic cycle involving oxidative addition, amido complex formation, and reductive elimination.

## 1. Introduction

Transition-metal-catalyzed arylation has become a cornerstone strategy for constructing C–N bonds in organic synthesis. Palladium catalysis is particularly effective due to its broad ligand compatibility, enabling efficient bond formation across diverse substrates. Beyond palladium, other transition metals such as rhodium, copper, iridium, cobalt, iron, and nickel have also been explored for arylation reactions [1,2,3,4,5,6]. Related *N*-arylation processes mediated by nickel, iron, copper, cobalt, and rhodium have likewise been documented [7,8,9,10,11]. In addition to its pivotal role in arylation reactions, palladium can also catalyze other transformations, such as alkylation [12], hydrogenation [13], hydroformylation [14], oxidation [15], intramolecular hydroamination [16], and cyclocarbonylation [17]. These examples demonstrate that palladium still holds substantial value for research and development in the field of organometallic catalysis. However, the application of palladium in Buchwald–Hartwig type reactions in aqueous environments remains underexplored. Some studies have demonstrated the feasibility of conducting palladium-catalyzed reactions in water, significantly enhancing the environmental sustainability and efficiency of these processes [18,19,20,21]. This shift towards aqueous media not only aligns with green chemistry principles but also opens new avenues for research and development in sustainable synthetic methodologies.

**Figure 1:**
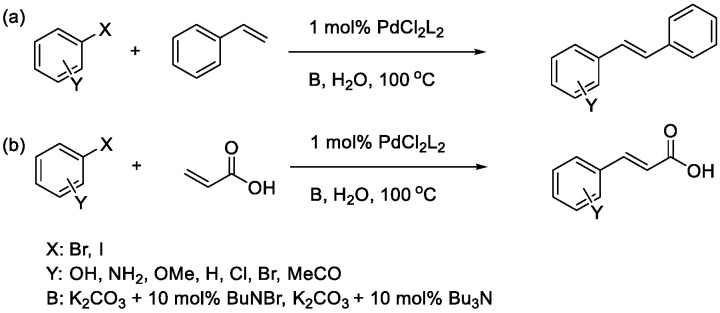
Example: palladium-catalyzed arylation of haloarenes with styrenes (**a**) or acrylic acids (**b**) in water [25].

From a green chemistry standpoint, performing organic reactions in water is increasingly attractive because water is inexpensive, non-toxic, and sometimes confers unique reactivity compared with conventional organic solvents [22,23,24]. Early examples of aqueous Pd-catalyzed arylation reactions illustrate the feasibility of high yield C–C bond-forming processes in water (Figure 1) [25].

**Figure 2:**
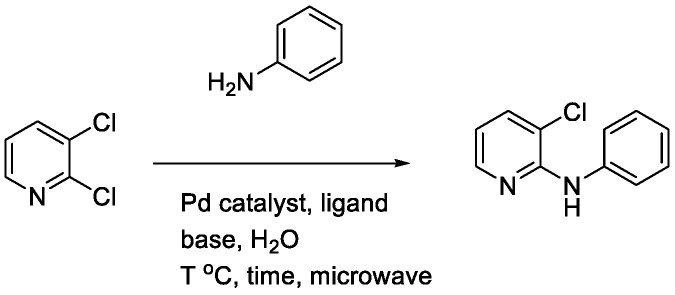
Example: Optimization of conditions for the palladium-catalyzed reaction of aniline with 2,3-dichloropyridine in water.

In addition to water, methanol and ethanol are also considered green solvents [26]. However, in terms of convenience, cost-effectiveness, and environmental friendliness, water has an overwhelming advantage over the latter two [27,28]. Accordingly, in our laboratory’s previous work on allylic reactions, we successfully employed transition-metal catalysts with water as the solvent and obtained products in high yields [29,30,31]. In parallel, microwave-assisted synthesis has emerged as a powerful enabling technology to accelerate reactions and improve reproducibility by rapid and homogeneous heating in sealed vessels [32]. Water demonstrates a high relative permittivity and substantial dielectric loss at microwave frequencies, thereby facilitating efficient coupling and expeditious heating within alternating electric fields. This renders it a more suitable microwave heating medium than most organic solvents [33,34]. Motivated by these developments and by precedent research for microwave-assisted *N*-arylation in toluene [35], we sought to develop a microwave-assisted Pd-catalyzed *N*-arylation in water for anilines with 2,3-dihalopyridines, aiming to improve operational simplicity and environmental footprint while maintaining high efficiency (Figure 2).

Building on these themes, we sought to merge aqueous reaction media with microwave assistance for palladium-catalyzed *N*-arylation between anilines and 2,3-dihalopyridines. Prior work using toluene under microwave irradiation achieved a 71% yield for the *N*-arylation of aniline with 2,3-dichloropyridine. We hypothesized that water could replace organic solvent while retaining or improving efficiency under carefully selected catalytic systems and bases.

Herein, we report an optimized, selective *N*-arylation of anilines with 2,3-dihalopyridines in water using a PdCl_2_(1,10-phenanthroline)_2_ [PdCl_2_(1,10-Phen)_2_]/BINAP catalyst combination and K_3_PO_4_ as a base under microwave heating. We detail the effects of catalyst/ligand ratios, temperature and time, base identity, palladium precursors, phosphine ligands, and substrate scopes, achieving up to 91% yield in 30 min at 150 °C. This method advances sustainable cross-coupling chemistry by leveraging water as the solvent and microwave technology to enhance atom economy and operational simplicity.

## 2. Materials and Methods

### 2.1. General Information

Microwave irradiation was performed using a focused microwave synthesizer (CEM Discover S-Class, CEM Corporation, Matthews, NC, USA). IR spectra were recorded on a PerkinElmer System 2000 FT-IR spectrophotometer (PerkinElmer Inc., Shelton, CT, USA). NMR spectra were acquired on Varian Unity-plus 400, Varian Unity-inova 500, or Varian Mercury-plus 400 instruments (Varian Inc., Palo Alto, CA, USA). GC–MS analysis was performed on a Thermo-Finnigan GC–MS system (Thermo Finnigan, Waltham, MA, USA). HRMS measurements were obtained on a Shimadzu QP2010 instrument (Shimadzu Corporation, Kyoto, Japan). Chemical shifts are reported in ppm (δ) relative to TMS as an internal standard; coupling constants (J) are reported in Hz.

### 2.2. General Procedure for Microwave-Assisted N-Arylation in Water

A sealed microwave vial was charged sequentially with 2,3-dihalopyridines **1** (1.0 mmol), anilines **2** (1.2 mmol), PdCl_2_(1,10-Phen)_2_ (0.02 mmol), (±)-BINAP (0.03 mmol), K_3_PO_4_ (3.5 mmol), and H_2_O (2.5 mL). The mixture was irradiated under microwave heating at 150 °C for 30 min. After cooling, the reaction mixture was extracted with dichloromethane. The organic layer was dried over anhydrous MgSO_4_, filtered, and concentrated under reduced pressure. The crude residue was purified by silica gel column chromatography (hexanes/EtOAc = 4:1) to afford the desired aminopyridine products.

## 3. Results

### 3.1. Reaction Optimization

To establish an efficient aqueous *N*-arylation, 2,3-dichloropyridine (**1a**) and aniline (**2a**) were selected as model substrates. Initial experiments using Pd(OAc)_2_ and (±)-BINAP in water under conventional heating afforded only modest yield (Table 1, entry 1). Microwave irradiation substantially increased yield while reducing reaction time (Table 1, entry 2), in line with the general advantages of microwave-assisted synthesis [32].

#### 3.1.1. Optimization of Catalyst and Ligand Ratios

Varying the Pd(OAc)_2_/(±)-BINAP ratio indicated that excess ligand relative to palladium improved the isolated yield, consistent with ligand-enabled stabilization and catalytic turnover under aqueous microwave conditions (Table 1). Notably, omission of either palladium or ligand dramatically reduced conversion (Table 1, entries 7–9), supporting a bona fide catalyzed process.

#### 3.1.2. Effects of Temperature, Time, and Stoichiometry

Optimization of reaction temperature and time revealed that 150 °C for 30 min delivered improved yield relative to 180 °C, whereas excessive heating (200 °C) or shorter reaction time reduced yield (Table 2). Altering the amount of aniline present did not further improve yields beyond the 1:1.2 ratio.

#### 3.1.3. Base Screening

A variety of bases were examined (Table 3). Among them, K_3_PO_4_ provided the highest isolated yield (85%) under otherwise identical conditions, and was selected for further studies. Reactions in the absence of a base resulted in diminished yield even upon extended irradiation.

#### 3.1.4. Palladium Sources and Ligand Effects

Commercially available palladium precursors were screened (Table 4), identifying PdCl_2_(1,10-Phen)_2_ as the most effective catalyst (91% isolated yield). Subsequently, phosphine ligands were evaluated with PdCl_2_(1,10-Phen)_2_ (Table 5). The bidentate ligand (±)-BINAP furnished the best outcome (91%), while many monodentate phosphines gave substantially lower yields. Collectively, these data defined the optimized conditions used for substrate scope evaluation.

### 3.2. Substrate Scope

#### 3.2.1. Variation in 2,3-Dihalopyridines

Using aniline (**2a**) as a coupling partner, various 2,3-dihalopyridines were evaluated (Table 6, Figures S1–S16). For symmetrical dihalides (same halogen at C2/C3), products were obtained with predominant coupling at C2 under the optimized conditions. For mixed halides, the major product generally corresponded to coupling at the position bearing the less electronegative halogen, reflecting the greater facility of oxidative addition into the weaker, more polarizable C–X bond at that site and thus the differential C–X bond activation tendencies early in the catalytic cycle [36].

#### 3.2.2. Variation in Anilines

A series of substituted anilines were coupled with 2,3-dichloropyridine (**1a**) (Table 7, Figures S17–S46). Both electron-donating and electron-withdrawing substituents were tolerated, affording moderate to excellent yields. A plausible explanation is that the strongly electron-withdrawing nitro substituents generally reduced yield, plausibly due to decreased nucleophilicity of the aniline nitrogen. Ortho-disubstitution also diminished yield, consistent with steric hindrance effects.

## 4. Discussion

This study aimed to develop an efficient and more sustainable protocol for the Pd-catalyzed *N*-arylation of anilines with 2,3-dihalopyridines. A systematic evaluation of the catalytic system and reaction parameters identified a set of conditions that consistently provided superior performance under microwave irradiation. Overall, the results demonstrate that the combination of PdCl_2_(1,10-Phen)_2_ and (±)-BINAP in the presence of an inorganic base, using water as the reaction medium and heating at 150 °C by microwave irradiation, constitutes the most effective platform for this transformation.

The choice of Pd source was found to be a decisive factor in determining both conversion and isolated yield. Among the Pd precatalysts examined, PdCl_2_(1,10-Phen)_2_ delivered the most consistently high yields, whereas Pd(OAc)_2_ remained competent. This difference suggests that the pre-coordinated phenanthroline-containing Pd complex may generate the catalytically active species more efficiently under the aqueous microwave conditions and/or exhibit improved stability against deactivation pathways in this medium.

Ligand loading and the Pd:(±)-BINAP ratio also exerted a pronounced influence. When the Pd:BINAP ratio was varied from 1:1 to 1:4, the best results were obtained with ratios between 1:2 and 1:4, indicating that an excess of BINAP relative to Pd is beneficial. Practically, this behavior is consistent with the need to maintain a sufficient population of ligated Pd species to sustain productive turnover and to suppress off-cycle processes that can arise under high-temperature conditions.

The identity of the base strongly affected reaction efficiency. In the base screen, the effectiveness followed the trend: K_3_PO_4_ > Cs_2_CO_3_ ≥ K_2_CO_3_ > NaOH.

Notably, K_3_PO_4_ emerged as the optimal base, delivering superior yields and cleaner conversions relative to the carbonate bases.

Microwave heating enabled rapid attainment of productive reaction temperatures and significantly shortened reaction times. A temperature of 150 °C with a reaction time of 30 min provided the optimal balance between high conversion and operational efficiency. Shorter reaction times under otherwise identical conditions led to incomplete conversion, indicating that a minimum residence time is required for full consumption of the dihalopyridine substrate and completion of the coupling process, even under intensified microwave conditions.

On the basis of the combined optimization results, the following conditions were selected as standard for subsequent studies—a molar ratio of 2,3-dichloropyridine (**1a**):aniline (**2a**) = 1:1.2, PdCl_2_(1,10-Phen)_2_ (0.02 mmol) as the catalyst, (±)-BINAP (0.03 mmol) as the ligand, and K_3_PO_4_ (3.5 mmol) as the base—conducted in water at 150 °C under microwave irradiation for 30 min. Using this optimized protocol, the *N*-arylation of various 2,3-dihalopyridines (**1**) with diverse anilines (**2**) was investigated. The reaction exhibited robust performance across the examined substrate set, highlighting the generality of the catalytic platform under the chosen conditions.

Solvent screening revealed that water is not merely a viable alternative medium, but in this system, provides advantages in both yield and sustainability. While methanol and ethanol could also support product formation, they were less favorable overall relative to water. Importantly, when benchmarked against reported toluene-based microwave protocols (approximately 71% yield), the present aqueous method delivered higher yields (up to 91%), required shorter reaction times, and improved the overall environmental profile of the process.

From a green chemistry perspective [37], the use of water reduces reliance on volatile and flammable organic solvents, thereby lowering safety risks and minimizing environmental impact. Moreover, employing water as the reaction medium aligns with waste-reduction principles by limiting auxiliary materials associated with solvent handling and disposal. Collectively, these features position the developed aqueous microwave protocol as a practical and more sustainable approach to Pd-catalyzed *N*-arylation of dihalopyridines with anilines.

## 5. Conclusions

In summary, this study establishes that PdCl_2_(1,10-Phen)_2_/(±)-BINAP in combination with K_3_PO_4_ under aqueous microwave conditions (150 °C, 30 min) constitutes an efficient and operationally attractive system for the *N*-arylation of 2,3-dihalopyridines with anilines. The method offers improved yield and reduced reaction time relative to toluene-based microwave approaches, while simultaneously advancing sustainability through the use of water as a benign solvent.

## Figures and Tables

**Table 1:** Reaction of 2,3-dichloropyridine (**1a**) with aniline (**2a**): effects of ratio of catalyst to ligand ^a^.

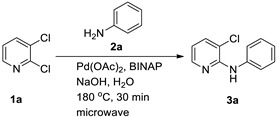		
Entry	Pd(OAc)_2_:BINAP	Yield (%) ^b^
1 ^c^	0.02:0.02	30
2	0.02:0.02	53
3	0.01:0.01	46
4	0.02:0.03	71
5	0.01:0.015	58
6	0.02:0.08	61
7	0:0.03	32
8	0.02:0	15
9	0:0	<1%

^a^ Reaction conditions: **1a** (1 mmol), **2a** (1.2 mmol), Pd(OAc)_2_, BINAP and NaOH (3.5 mmol) were reacted in water (2.5 mL) under microwave irradiation at 180 °C for 30 min. ^b^ Isolated yield. ^c^ Reflux for 3 h.

**Table 2:** Reaction of 2,3-dichloropyridine (**1a**) with aniline (**2a**): effects of temperature and reaction time and ratio of reactant ^a^.

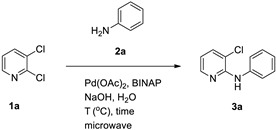			
Entry	Time (min)	T (°C)	Yield (%) ^b^
1	10	180	62
2	15	180	65
3	30	180	71
4	60	180	69
5	30	100	77
6	30	150	81
7	30	200	61
8 ^c^	30	150	72
9 ^d^	30	150	74

^a^ Reaction conditions: **1a** (1 mmol), **2a** (1.2 mmol), Pd(OAc)_2_ (0.02 mmol), BINAP (0.03 mmol) and NaOH (3.5 mmol) were reacted in water (2.5 mL) under microwave irradiation. ^b^ Isolated yield. ^c^ Compound **2a** (0.8 mmol) was used. ^d^ Compound **2a** (2 mmol) was used.

**Table 3:** Reaction of 2,3-dichloropyridine (**1a**) with aniline (**2a**): effects of base ^a^.

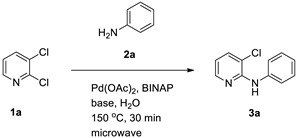		
Entry	Base	Yield (%) ^b^
1	K_2_CO_3_	75
2	K_3_PO_4_	85
3	Cs_2_CO_3_	78
4	Na_2_CO_3_	75
5	NaHCO_3_	61
6	KOH	77
7	NaOH	81
8	-	50
9 ^c^	-	27

^a^ Reaction conditions: **1a** (1 mmol), **2a** (1.2 mmol), Pd(OAc)_2_ (0.02 mmol), BINAP (0.03 mmol), and base (3.5 mmol) were reacted in water (2.5 mL) under microwave irradiation at 150 °C for 30 min. ^b^ Isolated yield. ^c^ Microwave for 2 h.

**Table 4:** Reaction of 2,3-dichloropyridine (**1a**) with aniline (**2a**): effects of palladium catalyst ^a^.

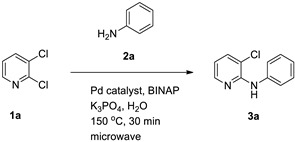		
Entry	Pd Catalyst	Yield (%) ^b^
1	Pd(OAc)_2_	85
2	Pd(acac)_2_	77
3	Pd(propionate)_2_	77
4	PdCl_2_	78
5	Pd(OCOCF_3_)_2_	80
6	PdCl_2_(CH_3_CN)_2_	80
7	PdCl_2_(PhCN)_2_	70
8	PdCl_2_(1,10-Phen)_2_	91
9	PdF_6_(acac)_2_	72
10	Pd_2_(dba)_3_	76
11	Pd(PPh_3_)_4_	55
12	[Pd(allyl)Cl]_2_	73

^a^ Reaction conditions: **1a** (1 mmol), **2a** (1.2 mmol), Pd catalyst (0.02 mmol), BINAP (0.03 mmol), and K_3_PO_4_ (3.5 mmol) were reacted in water (2.5 mL) under microwave irradiation at 150 °C for 30 min. ^b^ Isolated yield.

**Table 5:** Reaction of 2,3-dichloropyridine (**1a**) with aniline (**2a**): effects of phosphine ligand ^a^.

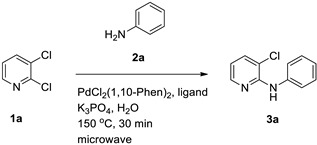		
Entry	Ligand	Yield (%) ^b^
1	PPh_3_	27
2	(4-ClC_6_H_4_)_3_P	11
3	(4-MeOC_6_H_4_)_3_P	20
4	[2,6-(MeO)_2_C_6_H_3_]_3_P	28
5	[2,4,6-(MeO)_3_C_6_H_2_]_3_P	45
6	(2-furyl)_3_P	40
7	(*p*-CH_3_C_6_H_4_)_3_P	45
8	(*o*-CH_3_C_6_H_4_)_3_P	19
9	Bu_3_P	6
10	(4-FC_6_H_4_)_3_P	50
11	TPPTS	50
12	(±)-BINAP	91
13 ^c^	(±)-BINAP	25
14	dppm ^d^	9
15	dppe ^d^	20
16	dppp ^d^	58
17	dppb ^d^	54
18	dpph ^d^	29
19	dppf ^d^	70

^a^ Reaction conditions: **1a** (1 mmol), **2a** (1.2 mmol), PdCl_2_(1,10-Phen)_2_ (0.02 mmol), ligand (0.03 mmol), and K_3_PO_4_ (3.5 mmol) were reacted in water (2.5 mL) under microwave irradiation at 150 °C for 30 min. ^b^ Isolated yield. ^c^ Reflux for 3 h. ^d^ 1,2-bis-(diphenylphosphino)ethane (dppe), 1,3-bis-(diphenylphosphino)propane (dppp), 1,4-bis-(diphenylphosphino)butane (dppb), 1,6-bis-(diphenylphosphino)hexane (dpph), and 1,1′-bis-(diphenylphosphino)ferrocene (dppf).

**Table 6:** Reaction of 2,3-dihalopyridines **1** with aniline (**2a**) under optimized conditions ^a^.

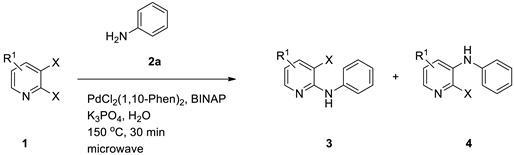					
Entry		1	Product		Yields (%) ^b^
1	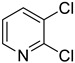	**1a**	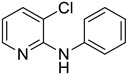	**3a**	91
2	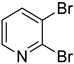	**1b**	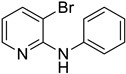	**3b**	36
3	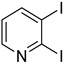	**1c**	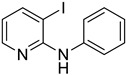	**3c**	8
4	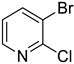	**1d**	**3b**		20
			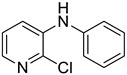	**4e**	28
5	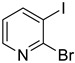	**1e**	**3c**		1
			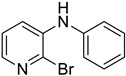	**4f**	35
6	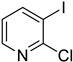	**1f**	**4e**		55
7	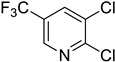	**1g**	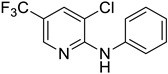	**3h**	70
8	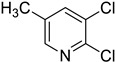	**1h**	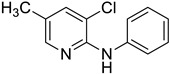	**3i**	86
9	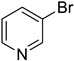	**1i**	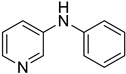	**5**	42

^a^ Reaction conditions: **1** (1 mmol), **2a** (1.2 mmol), PdCl_2_(1,10-Phen)_2_ (0.02 mmol), BINAP (0.03 mmol), and K_3_PO_4_ (3.5 mmol) were reacted in water (2.5 mL) under microwave irradiation at 150 °C for 30 min. ^b^ Isolated yield.

**Table 7:** Reaction of 2,3-dichloropyridine (**1a**) with anilines **2** under optimized conditions ^a^.

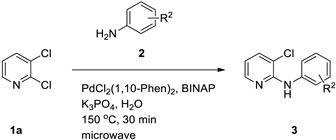				
Entry	2	R^2^	Product	Yield (%) ^b^
1	**2b**	2-Me	**6**	86
2	**2c**	2-Cl	**7**	77
3	**2d**	2-NO_2_	**8**	38
4	**2e**	2-Me, 4-Cl	**9**	82
5	**2f**	2,4-Di-Me	**10**	90
6	**2g**	2-Cl, 4-Me	**11**	88
7	**2h**	2,6-Di-Me	**12**	39
8	**2i**	3-Me	**13**	76
9	**2j**	3-OCH_2_Ph	**14**	80
10	**2k**	3-NO_2_	**15**	68
11	**2l**	3,5-Di-OMe	**16**	81
12	**2m**	3,5-Di-NO_2_	**-**	0
13	**2n**	4-Me	**17**	88
14	**2o**	4-OMe	**18**	94
15	**2p**	4-Cl	**19**	70
16	**2q**	4-NO_2_	**20**	62

^a^ Reaction conditions: **1** (1 mmol), **2a** (1.2 mmol), PdCl_2_(1,10-Phen)_2_ (0.02 mmol), BINAP (0.03 mmol), and K_3_PO_4_ (3.5 mmol) were reacted in water (2.5 mL) under microwave irradiation at 150 °C for 30 min. ^b^ Isolated yield.

## Data Availability

The original contributions presented in this study are included in the article/Supplementary Material. Further inquiries can be directed towards the corresponding author.

## Supplementary Materials

The following supporting information can be downloaded at https://www.mdpi.com/article/10.3390/ma19051003/s1, Figure S1: ^1^H NMR of **3a**; Figure S2: ^13^C NMR of **3a**; Figure S3: ^1^H NMR of **3b**; Figure S4: ^13^C NMR of **3b**; Figure S5: ^1^H NMR of **3c**; Figure S6: ^13^C NMR of **3c**; Figure S7: ^1^H NMR of **4e**; Figure S8: ^13^C NMR of **4e**; Figure S9: ^1^H NMR of **4f**; Figure S10: ^13^C NMR of **4f**; Figure S11: ^1^H NMR of **3h**; Figure S12: ^13^C NMR of **3h**; Figure S13: ^1^H NMR of **3i**; Figure S14: ^13^C NMR of **3i**; Figure S15: ^1^H NMR of **5**; Figure S16: ^13^C NMR of **5**; Figure S17: ^1^H NMR of **6**; Figure S18: ^13^C NMR of **6**; Figure S19: ^1^H NMR of **7**; Figure S20: ^13^C NMR of **7;** Figure S21: ^1^H NMR of **8**; Figure S22: ^13^C NMR of **8**; Figure S23: ^1^H NMR of **9**; Figure S24: ^13^C NMR of **9**; Figure S25: ^1^H NMR of **10**; Figure S26: ^13^C NMR of **10**; Figure S27: ^1^H NMR of **11**; Figure S28: ^13^C NMR of **11**; Figure S29: ^1^H NMR of **12**; Figure S30: ^13^C NMR of **12**; Figure S31: ^1^H NMR of **13**; Figure S32: ^13^C NMR of **13**; Figure S33: ^1^H NMR of **14**; Figure S34: ^13^C NMR of **14**; Figure S35: ^1^H NMR of **15**; Figure S36: ^13^C NMR of **15**; Figure S37: ^1^H NMR of **16**; Figure S38: ^13^C NMR of **16**; Figure S39: ^1^H NMR of **17**; Figure S40: ^13^C NMR of **17;** Figure S41: ^1^H NMR of **18**; Figure S42: ^13^C NMR of **18;** Figure S43: ^1^H NMR of **19**; Figure S44: ^13^C NMR of **19**; Figure S45: ^1^H NMR of **20**; Figure S46: ^13^C NMR of **20**.
