# Supplementary material for: Efficient Microwave-Assisted Palladium-Catalyzed Selective N-Arylation of Anilines with 2,3-Dihalopyridines in Water
Source: Materials (Basel). 2026 Mar 5;19(5):1003. doi: 10.3390/ma19051003 (PMC12986283; doi:10.3390/ma19051003)
Supplement: Supplementary file 1 [file materials-19-01003-s001.zip › materials-4146834-supplementary.pdf]

Efficient Microwave-Assisted Palladium-Catalyzed Selective N-Arylation of Anilines with 2,3-Dihalopyridines in Water

Hao-Chun Hu [1], Cheng-Yi Chen [2] and Shyh-Chyun Yang [1,2,3,4,*]

[1] Department of Fragrance and Cosmetic Science, College of Pharmacy, Kaohsiung Medical University, Kaohsiung 807378, Taiwan; drjcount@livemail.tw

[2] School of Pharmacy, College of Pharmacy, Kaohsiung Medical University, Kaohsiung 807378, Taiwan; chengyi716@gmail.com

[3] Department of Medical Research, Kaohsiung Medical University Hospital, Kaohsiung 80756, Taiwan

[4] School of Pharmacy, College of Pharmacy, Taipei Medical University, Taipei 11031, Taiwan

* Correspondence: scyang@kmu.edu.tw

The Chemical Suppliers

Acros: palladium(II) chloride,bis(benzonitrile)palladium chloride, tri(2-furyl)phosphine, 3-nitroaniline,2-nitroaniline, bis(diphenylphosphino)-methane, 2,3-dichloro-5-(trifluoromethyl)pyridine, and 4-chloro-2-methylaniline.; **Alfa Aesar**: palladium(II)trifluoroacetate,triphenylphosphine, tris(4-chlorophenyl)phosphine,tris(o-tolyl)phosphine, 2,3-dichloropyridine, 2,3-dibromopyridine, 3-bromo-2-chloropyridine, 2-chloro-3-iodopyridine, and 2,6-dimethylaniline.; **Aldrich**: palladium(II) acetate, palladium(II) acetylacetonate, palladium(II)propionate, bis(acetonitrile)dichloro-palladium(II), dichloro(1,10-phenanthroline)palladium(II), palladium(II)-hexafluoroacetylacetonate, diphenyl(2-pyridyl)phosphine, tris(2,6-dimethoxyphenyl)phosphine, 2-chloroaniline, tris(2,4,6-trimethoxyphenyl)phosphine, tris(p-tolyl)phosphine, 3-benzyloxyaniline, 2-chloro-4-methylaniline, 4-chloroaniline, 1,2-bis-(diphenylphosphino)ethane, 1,3-bis-(diphenylphosphino)propane, 1,4-bis-(diphenylphosphino)butane, 1,6-bis-(diphenylphosphino)hexane, 2-bromo-3-iodopyridine, and 3-bromopyridine.; **Fluka**: (rac)-2,2′-bis(diphenylphosphino)-1,1′- and triphenylphosphine-3,3′,3′′-trisulfonic acid trisodium salt hydrate.; **Ferak**: o-toluidine.; **Lancaster**: tris(dibenzylidieneacetone)dipalladium(0), tris-(4-methoxyphenyl)-phosphine, tris-(4-fluorophenyl)phosphine, 3,5-dimethoxyaniline, and potassium phosphate.; **Matrix**: 2,3-diiodopyridine and

2,3-dichloro-5-methylpyridine.; **NOAH**: cesium carbonate.; **OSAKA**: potassium hydroxide.; **Riedel-de Haën**: aniline and potassium carbonate.; **STREM**: 1,1′-bis(diphenylphosphino)ferrocene.; **Tokyo Chemical Industry Co.**: tetrakis(triphenylphosphine) palladium(0), tri-*n*-butylphosphine, 4-nitroaniline, *p*-toluidine, 3,5-dinitroaniline, *p*-anisidine, *m*-toluidine, 2,4-dimethylaniline, sodium carbonate, and sodium hydrogen carbonate.

Representative Experimental Procedure and Characterization

Example: Synthesis of 3-Chloro-N-phenylpyridin-2-amine (3a)

2,3-Dichloropyridine (**1a**) (148 mg, 1.0 mmol), aniline (**2a**) (112 mg, 1.2 mmol), $PdCl_2(1,10\text{-Phen})_2$ (7 mg, 0.02 mmol), (±)-BINAP (19 mg, 0.03 mmol), K_3PO_4 (743 mg, 3.5 mmol), and H_2O (2.5 mL) were added sequentially to a sealed microwave vial. The vial was heated at 150°C for 30 min under microwave irradiation. After cooling, the mixture was transferred to a separatory funnel, brine was added, and the mixture was extracted with dichloromethane. The organic extract was dried over anhydrous $MgSO_4$, filtered, and concentrated. The crude residue was purified by silica gel column chromatography (hexanes/EtOAc = 4:1) to afford **3a** as an oil.

Representative Characterization Data

3-Chloro-N-phenylpyridin-2-amine (3a)

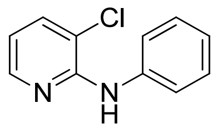

IR (KBr) ν 3407 cm^{-1}. ^1H NMR (500 MHz, $CDCl_3$) δ 6.68 (dd, J = 5.0, 7.5 Hz, 1H), 6.96 (br s, 1H, NH), 7.04 (t, J = 7.5 Hz, 1H), 7.33 (dd, J = 7.5, 8.5 Hz, 2H), 7.54 (dd, J = 1.5, 7.5 Hz, 1H), 7.62 (d, J = 8.5 Hz, 2H), 8.11 (dd, J = 1.5, 5.0 Hz, 1H). ^{13}C NMR (125 MHz, $CDCl_3$) δ 115.1, 116.0, 119.9, 122.7, 128.9, 136.6, 139.6, 145.7, 151.2. EI-MS m/z: 206 (M+2), 204 (M+), 168, 140, 115, 85, 77, 51. EI-HRMS (m/z) calcd for $C_{11}H_9ClN_2$: 204.0454; found: 204.0454.

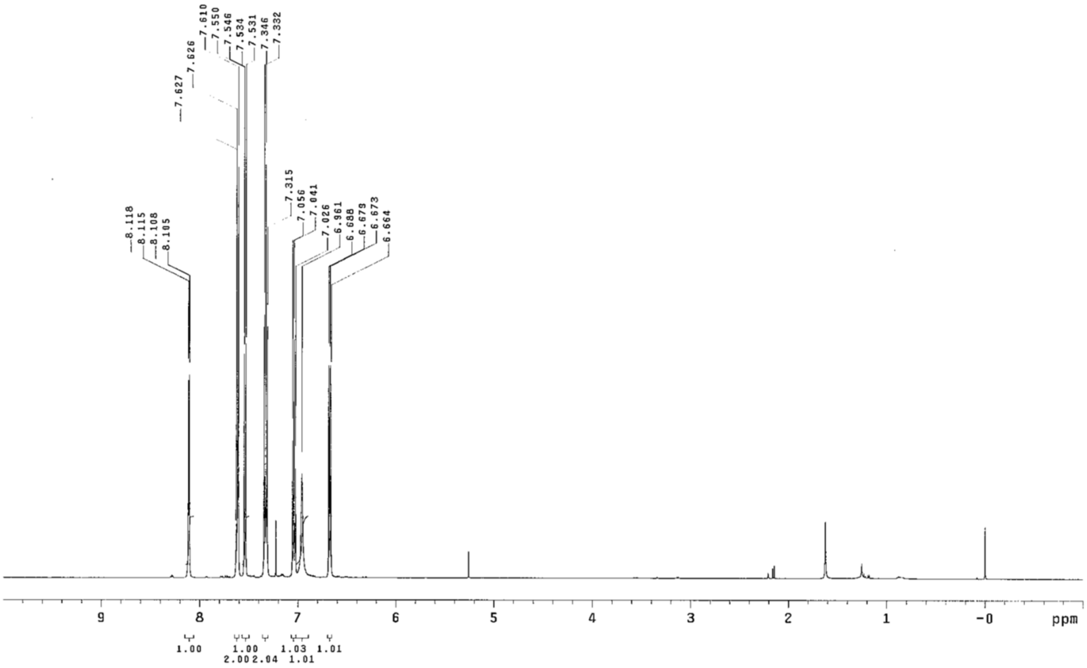

Figure S1: ^{1}H NMR of **3a**

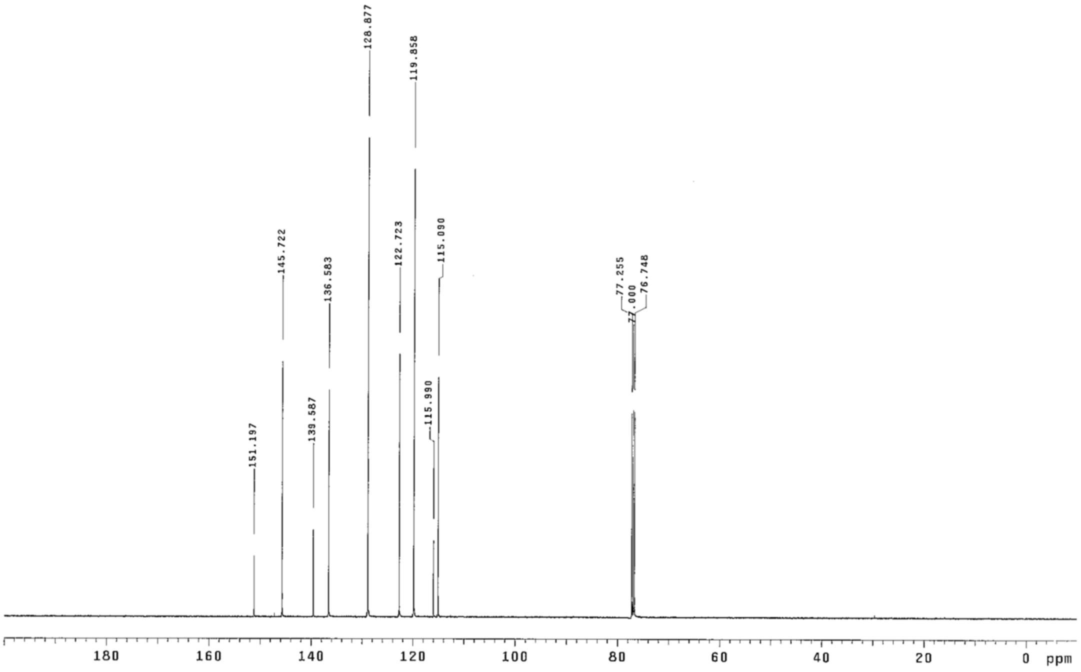

Figure S2: ^{13}C NMR of **3a**

3-Bromo-N-phenylpyridin-2-amine (3b)

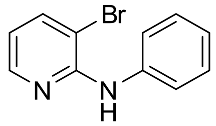

IR (KBr) ν 3397 cm^{-1}. ^1H NMR (500 MHz, $CDCl_3$) δ 6.61 (dd, J = 5.0, 8.0 Hz, 1H), 6.99 (br s, 1H, NH), 7.05 (dt, J = 1.5, 7.5 Hz, 1H), 7.34 (dd, J = 7.5, 8.5 Hz, 2H), 7.61 (dd, J = 1.5, 8.5 Hz, 2H), 7.71 (dd, J = 1.5, 8.0 Hz, 1H), 8.14 (dd, J = 1.5, 5.0 Hz, 1H). ^{13}C NMR (125 MHz, $CDCl_3$) δ 106.3, 115.6, 120.0, 122.8, 128.9, 139.7, 140.2, 146.4, 151.8. EI-MS m/z: 250 (M+2), 248 (M+), 183, 168, 140, 115, 85, 77, 63, 51. EI-HRMS (m/z) calcd for $C_{11}H_9BrN_2$: 247.9949; found: 247.9949.

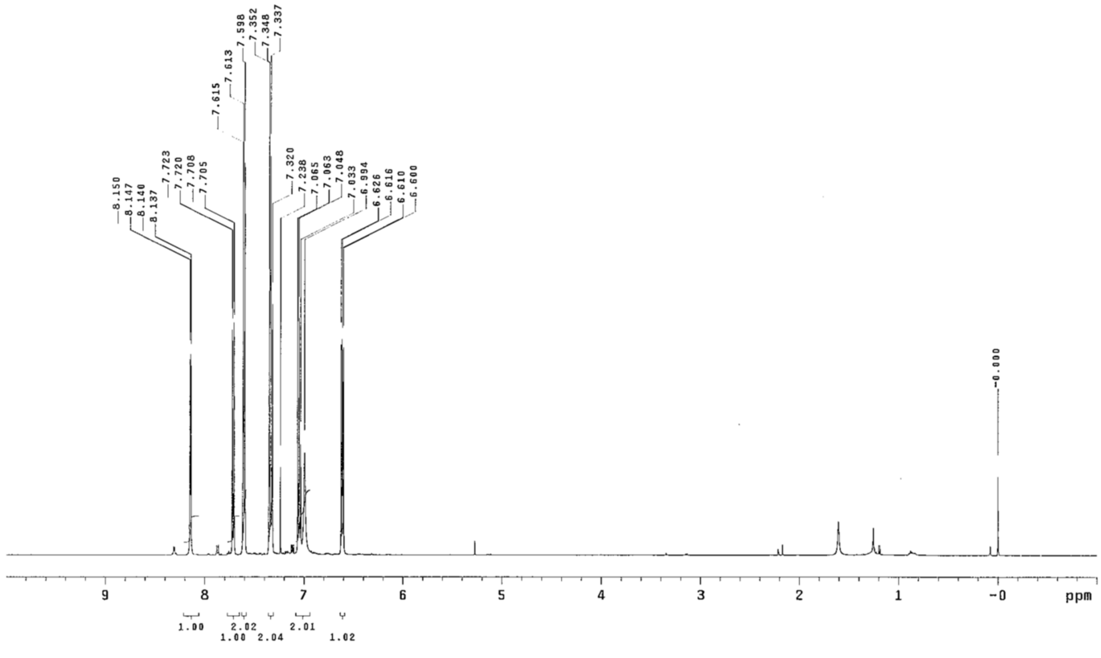

Figure S3: ^1H NMR of **3b**

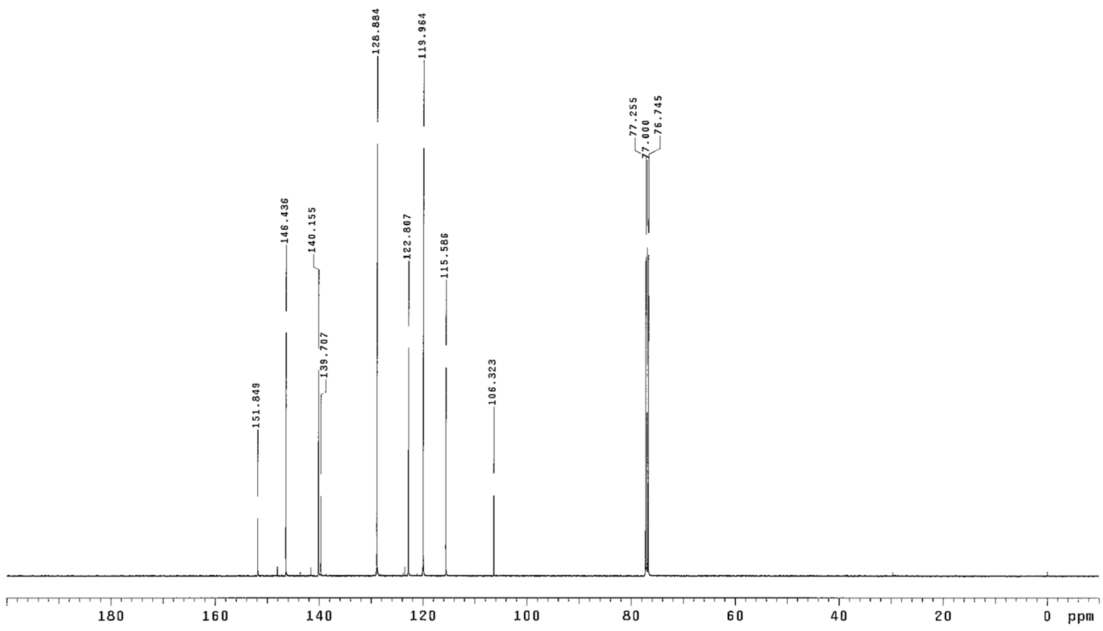

Figure S4: ^{13}C NMR of **3b**

3-Iodo-N-phenylpyridin-2-amine (3c)

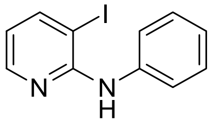

IR (KBr) ν 3388 cm^{-1}. ^{1}H NMR (400 MHz, $CDCl_3$) δ 6.48 (dd, J = 4.8, 7.6 Hz, 1H), 6.89 (br s, 1H, NH), 7.04 (t, J = 7.6 Hz, 1H), 7.34 (dd, J = 7.6, 8.4 Hz, 2H), 7.57 (d, J = 8.4 Hz, 2H), 7.93 (dd, J = 1.6, 7.6 Hz, 1H), 8.14 (dd, J = 1.6, 4.8 Hz, 1H). ^{13}C NMR (100 MHz, $CDCl_3$) δ 104.8, 116.2, 120.0, 122.9, 128.9, 140.0, 147.1, 147.4, 153.7. EI-MS m/z: 295 (M+), 204, 168, 141, 115, 89, 77, 63, 51. EI-HRMS (m/z) calcd for $C_{11}H_9IN_2$: 295.9810; found: 295.9808.

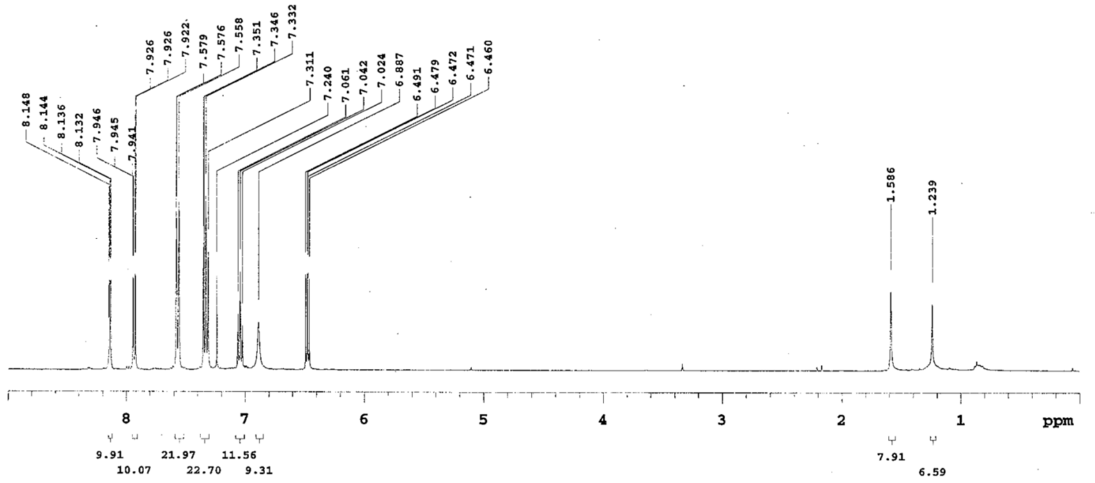

Figure S5: ^{1}H NMR of **3c**

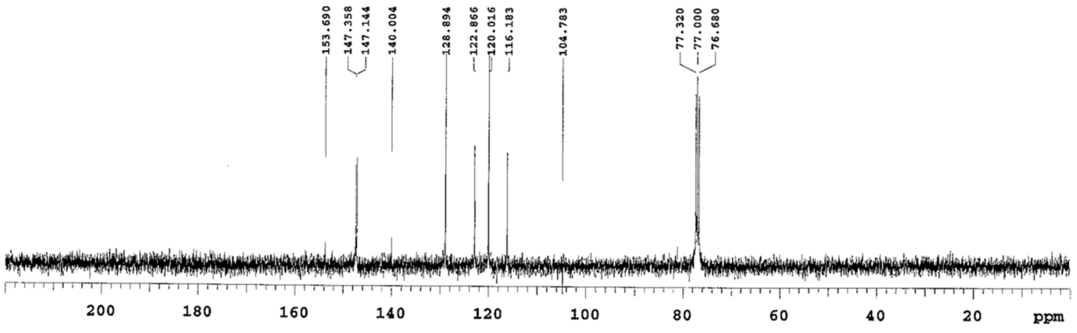

Figure S6: ^{13}C NMR of **3c**

2-Chloro-N-phenylpyridin-3-amine (4e)

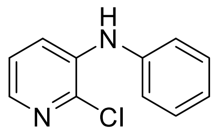

IR (KBr) ν 3388 cm^{-1}. ^{1}H NMR (400 MHz, $CDCl_3$) δ 6.14 (br s, 1H, NH), 7.04 (dd, J = 4.4, 8.0 Hz, 1H), 7.08 (t, J = 7.2 Hz, 1H), 7.12 (d, J = 8.4 Hz, 2H), 7.32 (dd, J = 7.2, 8.4 Hz, 2H), 7.45 (dd, J = 1.6, 8.0 Hz, 1H), 7.82 (dd, J = 1.6, 4.4 Hz, 1H). ^{13}C NMR (100 MHz, $CDCl_3$) δ 120.9, 121.1, 122.9, 123.7, 129.5, 137.5, 138.4, 139.1, 139.9. EI-MS m/z: 206 (M+2), 204 (M+), 169, 141, 114, 89, 85, 77, 63, 51. EI-HRMS (m/z) calcd for $C_{11}H_9ClN_2$: 204.0454; found: 204.0452.

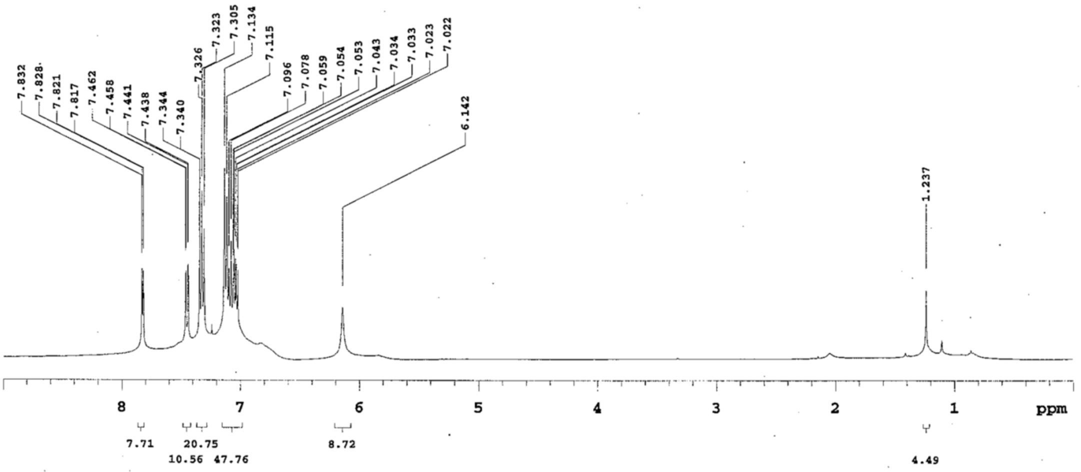

Figure S7: ^{1}H NMR of **4e**

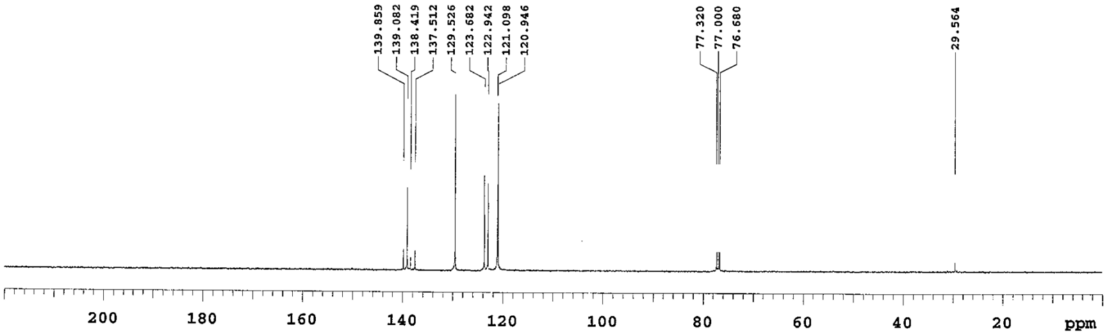

Figure S8: ^{13}C NMR of **4e**

2-Bromo-N-phenylpyridin-3-amine (4f)

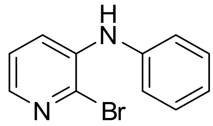

IR (KBr) ν 3378 cm^{-1}. ^{1}H NMR (400 MHz, $CDCl_3$) δ 6.16 (br s, 1H), 7.09 (dd, J = 4.4, 8.4 Hz, 1H), 7.12 (tt, J = 1.2, 8.4 Hz, 1H), 7.16 (dd, J = 1.2, 7.6 Hz, 2H), 7.36 (dd, J = 7.2, 8.4 Hz, 2H), 7.43 (dd, J = 1.6, 8.4 Hz, 1H), 7.84 (dd, J = 1.6, 4.4 Hz, 1H). ^{13}C NMR (100 MHz, $CDCl_3$) δ 121.1, 121.2, 123.3, 123.9, 129.7, 131.7, 139.3, 139.8, 139.9. EI-MS m/z: 250 (M+2), 248 (M+), 168, 140, 115, 84, 77, 63, 51. EI-HRMS (m/z) calcd for $C_{11}H_9BrN_2$: 247.9949; found: 247.9947.

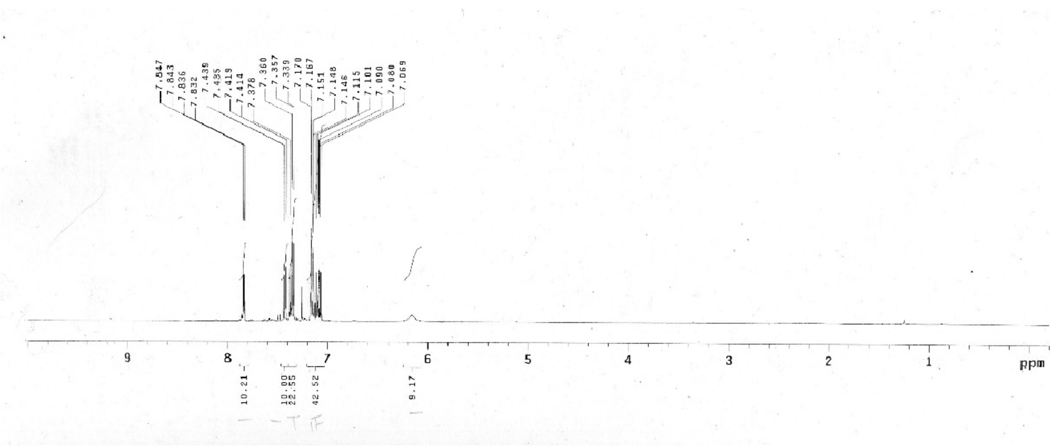

Figure S9: ^{1}H NMR of **4f**

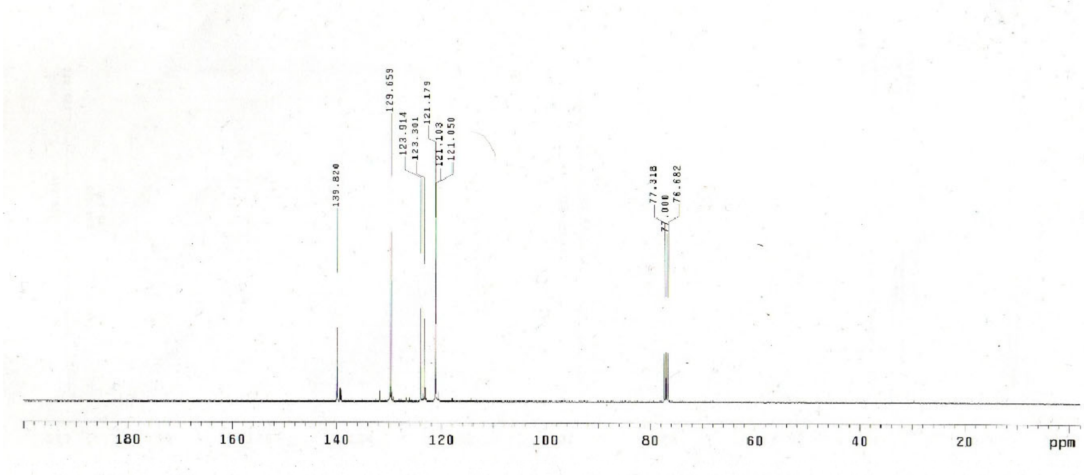

Figure S10: ^{13}C NMR of **4f**

3-Chloro-N-phenyl-5-(trifluoromethyl)pyridin-2-amine (3h)

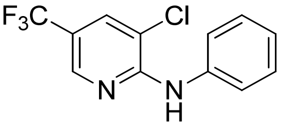

IR (KBr) ν 3244 cm^{-1}. ^{1}H NMR (400 MHz, $CDCl_3$) δ 7.13 (tt, J = 0.8, 7.6 Hz, 1H), 7.20 (br s, 1H, NH), 7.37 (dd, J = 7.6, 8.4 Hz, 2H), 7.61 (dd, J = 0.8, 8.4 Hz, 2H), 7.75 (d, J = 2.0 Hz, 1H), 8.36 (d, J = 2.0 Hz, 1H). ^{13}C NMR (100 MHz, $CDCl_3$) δ 115.6, 118.0, 120.8, 124.0, 124.7, 129.0, 133.4, 138.4, 143.5, 153.2. EI-MS m/z: 274 (M+2), 272 (M+), 253, 236, 217, 197, 168, 140, 118, 114, 77, 69, 51. EI-HRMS (m/z) calcd for $C_{12}H_8ClF_3N_2$: 272.0328; found: 272.0331.

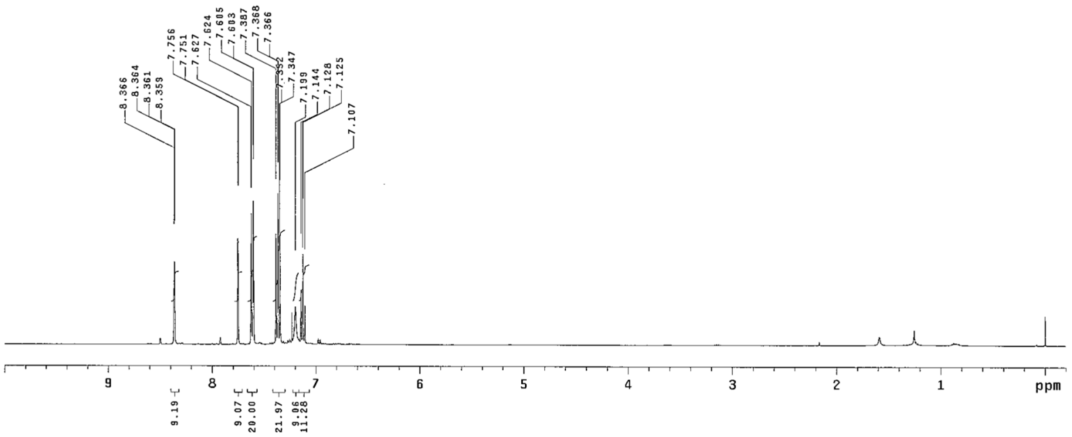

Figure S11: ^{1}H NMR of **3h**

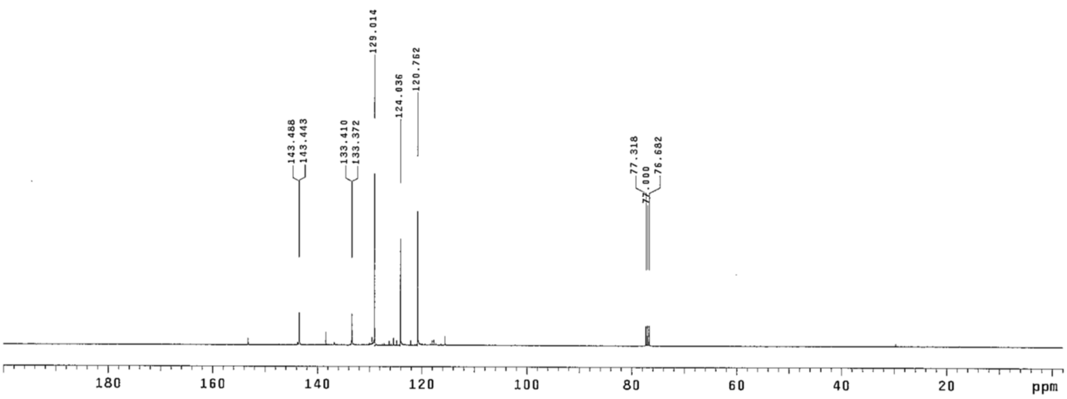

Figure S12: ^{13}C NMR of **3h**

3-Chloro-5-methyl-N-phenylpyridin-2-amine (3i)

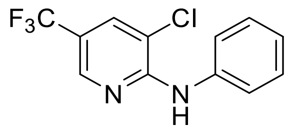

IR (KBr) ν 3397 cm^{-1}. ^{1}H NMR (500 MHz, $CDCl_3$) δ 2.24 (s, 3H, CH_3), 6.93 (br s, 1H, NH), 7.05 (t, J = 7.5 Hz, 1H), 7.35 (t, J = 7.5 Hz, 2H), 7.45 (d, J = 1.5 Hz, 1H), 7.60 (d, J = 7.5 Hz, 2H), 7.97 (d, J = 1.5 Hz, 1H). ^{13}C NMR (125 MHz, $CDCl_3$) δ 17.2, 115.9, 119.6, 122.5, 124.7, 128.9, 137.8, 139.8, 145.0, 149.1. EI-MS m/z: 220 (M+2), 218 (M+), 182, 168, 154, 127, 92, 77, 63, 51. EI-HRMS (m/z) calcd for $C_{12}H_{11}ClN_2$: 218.0611; found: 218.0609.

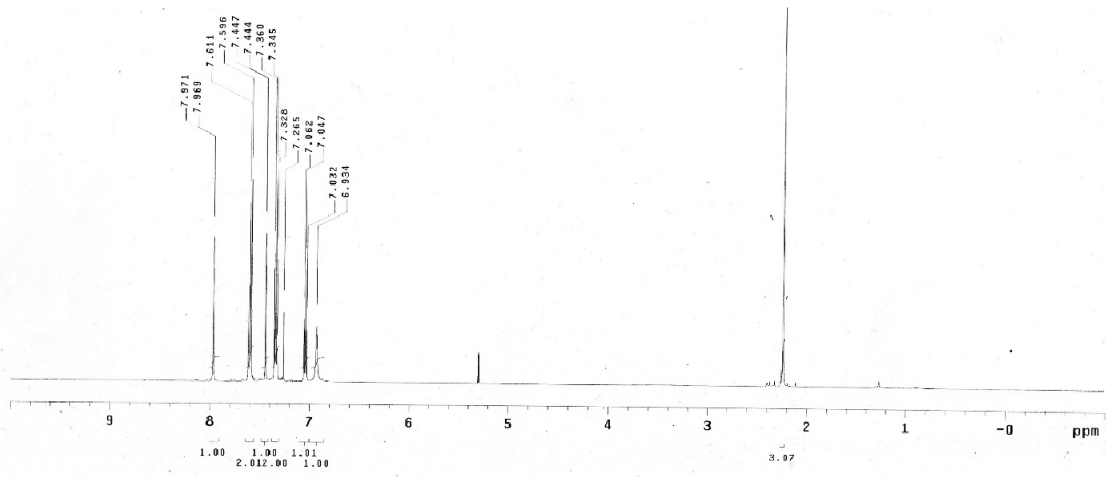

Figure S13: ^{1}H NMR of **3i**

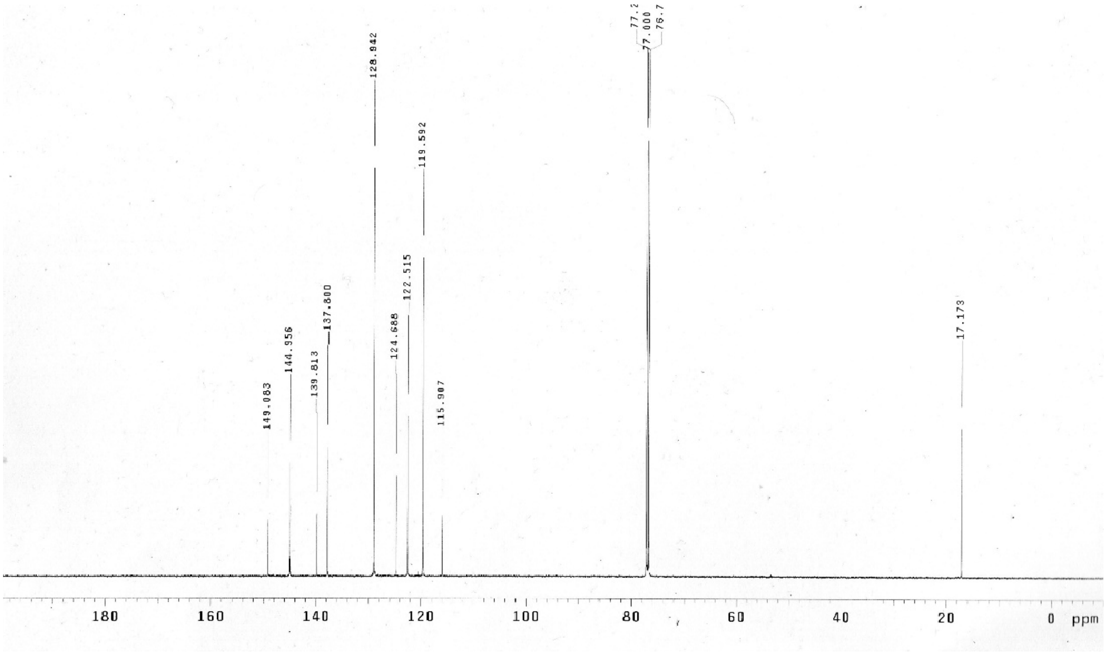

Figure S14: ^{13}C NMR of **3i**

N-Phenylpyridin-3-amine (5)

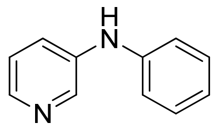

IR (KBr) ν 3397 cm^{-1}. ^{1}H NMR (500 MHz, $CDCl_3$) δ 5.85 (br s, 1H, NH), 7.00 (t, J = 7.5 Hz, 1H), 7.10 (d, J = 8.5 Hz, 2H), 7.18 (dd, J = 4.5, 8.0 Hz, 1H), 7.30 (dd, J = 7.5, 8.5 Hz, 2H), 7.42 (ddd, J = 1.0, 2.5, 8.0 Hz, 1H), 8.15 (dd, J = 1.0, 4.5 Hz, 1H),

8.38 (d, J = 2.5 Hz, 1H). ^{13}C NMR (125 MHz, $CDCl_3$) δ 118.4, 122.1, 123.5, 123.7, 129.5, 139.9, 139.9, 141.6, 141.8. EI-MS m/z: 170 (M+), 142, 115, 89, 77, 65, 51. EI-HRMS (m/z) calcd for $C_{11}H_{10}N_2$: 170.0844; found: 170.0847.

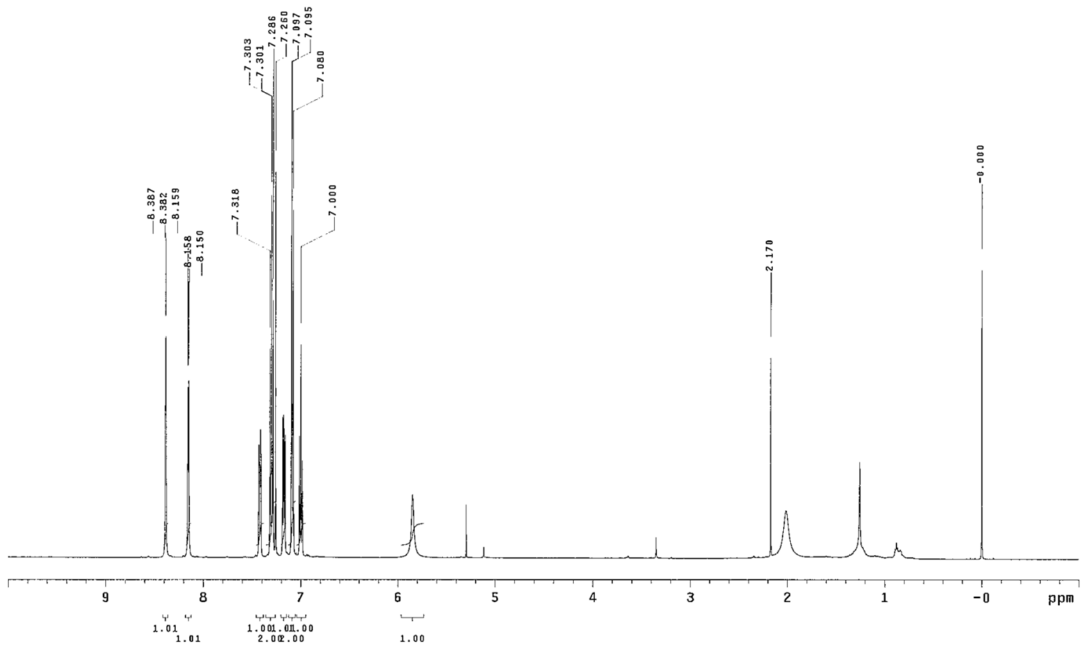

Figure S15: ^{1}H NMR of **5**

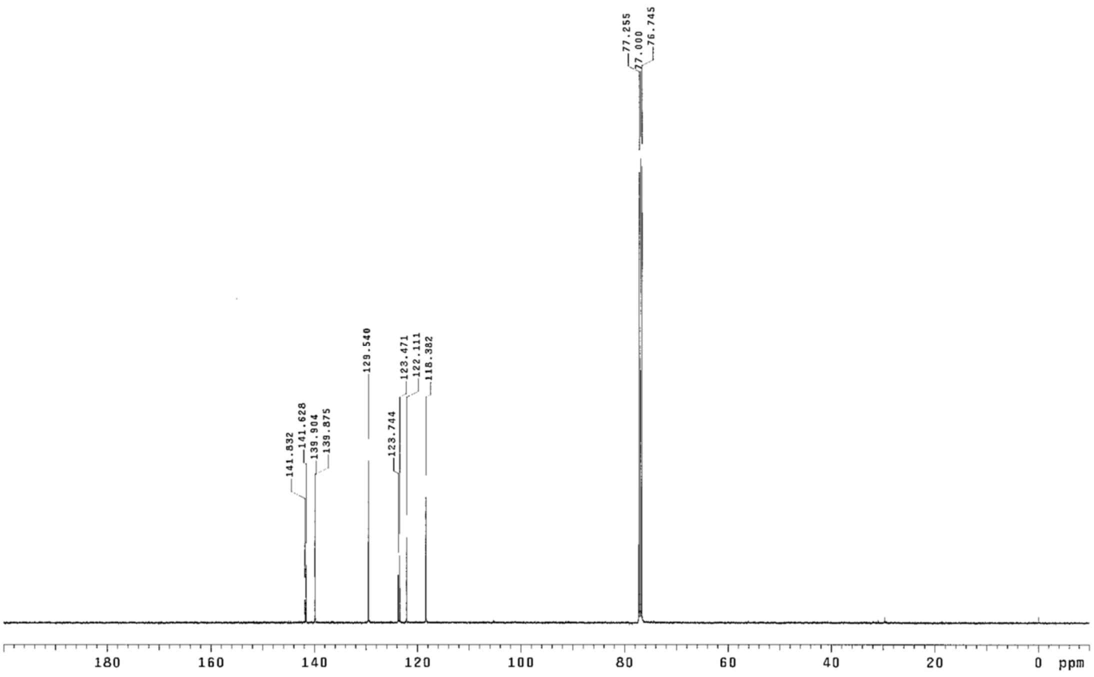

Figure S16: ^{13}C NMR of **5**

3-Chloro-N-(o-tolyl)pyridin-2-amine (6)

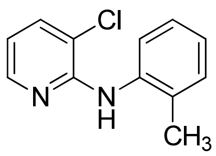

IR (KBr) ν 3426 cm^{-1}. ^1H NMR (500 MHz, $CDCl_3$) δ 2.31 (s, 3H, CH_3), 6.66 (dd, J = 5.0, 7.5 Hz, 1H), 6.82 (br s, 1H, NH), 7.04 (t, J = 7.5 Hz, 1H), 7.22 (d, J = 7.5 Hz, 1H), 7.25 (t, J = 7.5 Hz, 1H), 7.56 (dd, J = 1.5, 7.5 Hz, 1H), 7.98 (d, J = 7.5 Hz, 1H), 8.09 (dd, J = 1.5, 5.0 Hz, 1H). ^{13}C NMR (125 MHz, $CDCl_3$) δ 17.9, 114.7, 116.2, 122.0, 123.7, 126.6, 129.1, 130.5, 136.6, 137.7, 145.8, 151.6. EI-MS m/z: 220 (M+2), 218 (M+), 203, 181, 168, 154, 140, 127, 114, 108, 91, 77, 63, 51. EI-HRMS (m/z) calcd for $C_{12}H_{11}ClN_2$: 218.0611; found: 218.0614.

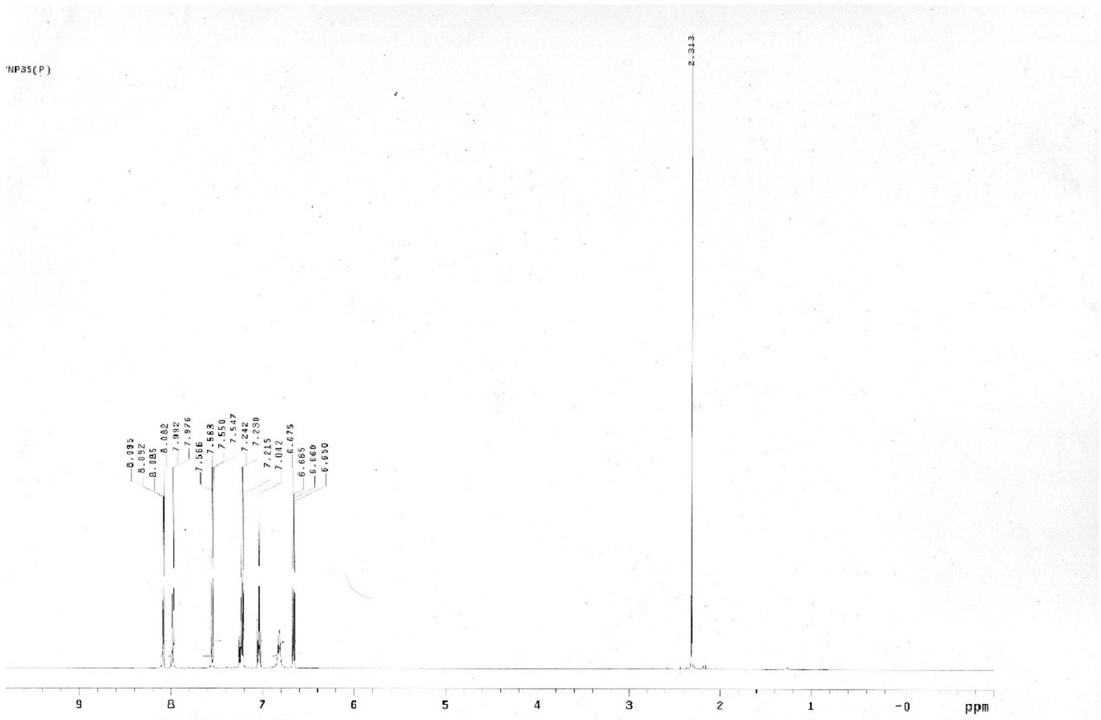

Figure S17: ^1H NMR of **6**

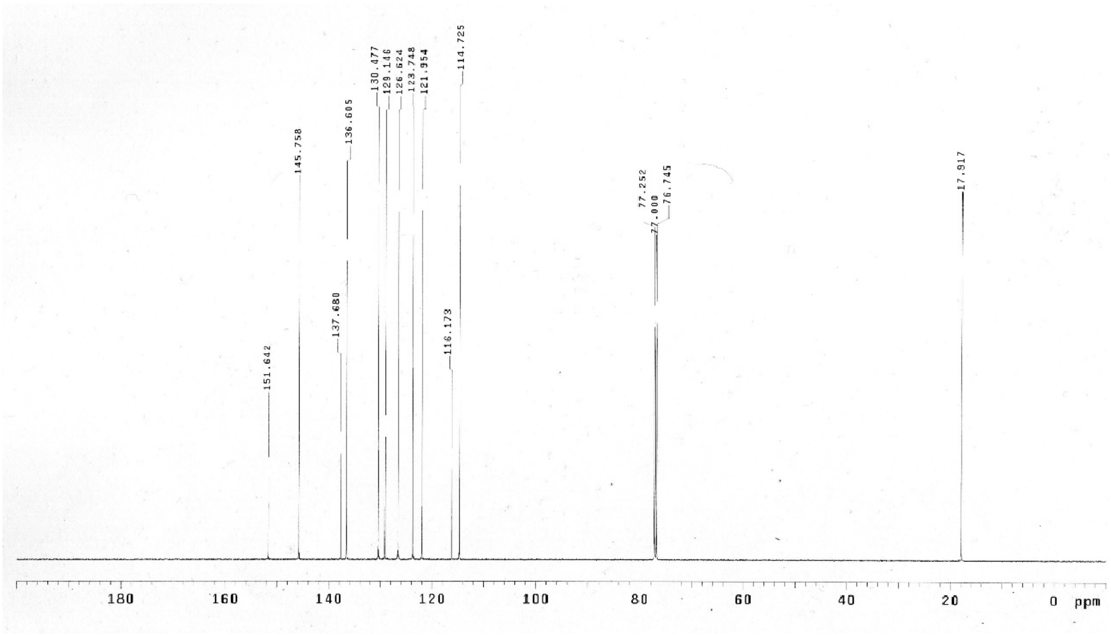

Figure S18: ^{13}C NMR of **6**

3-Chloro-N-(2-chlorophenyl)pyridin-2-amine (7)

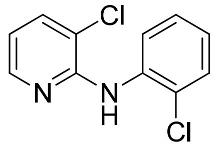

IR (KBr) ν 3388 cm^{-1}. ^{1}H NMR (500 MHz, $CDCl_3$) δ 6.80 (dd, J = 5.0, 8.0 Hz, 1H), 6.99 (ddd, J = 1.5, 7.5, 8.0 Hz, 1H), 7.31 (ddd, J = 1.5, 7.5, 8.5 Hz, 1H), 7.42 (dd, J = 1.5, 8.0 Hz, 1H), 7.65 (dd, J = 1.5, 8.0 Hz, 1H), 7.79 (br s, 1H, NH), 8.17 (dd, J = 1.5, 5.0 Hz, 1H), 8.57 (dd, J = 1.5, 8.5 Hz, 1H). ^{13}C NMR (125 MHz, $CDCl_3$) δ 115.8, 117.4, 120.7, 123.2, 123.4, 127.5, 129.2, 136.1, 137.5, 144.7, 150.6. EI-MS m/z: 242 (M+4), 240 (M+2), 238 (M+), 203, 168, 140, 115, 101, 89, 84, 75, 63, 50. EI-HRMS (m/z) calcd for $C_{11}H_8Cl_2N_2$: 238.0065; found: 238.0067.

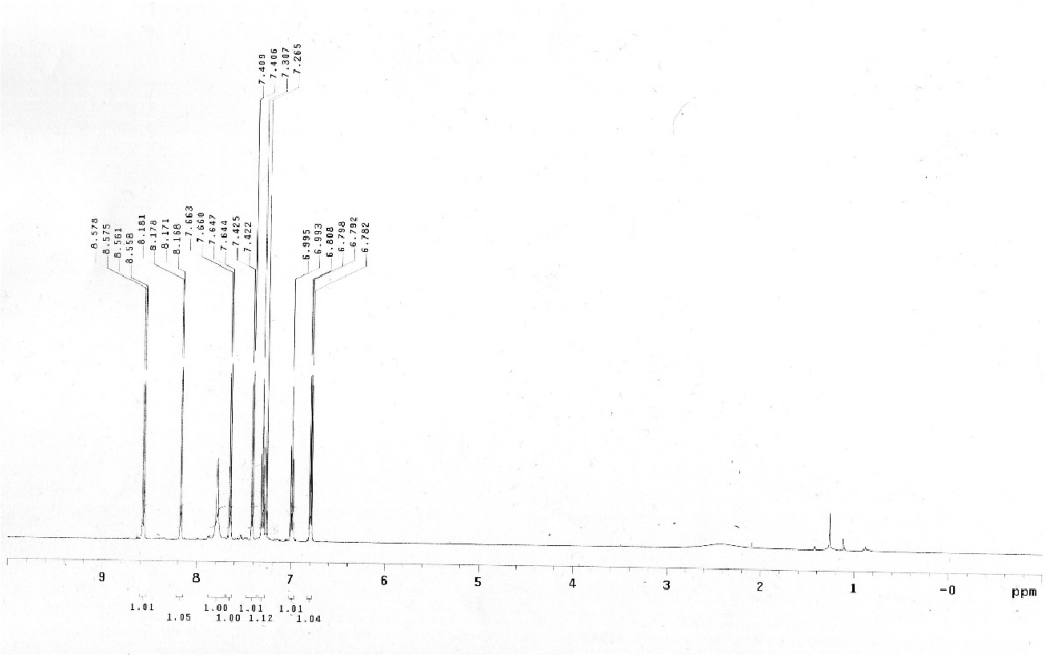

Figure S19: ^{1}H NMR of **7**

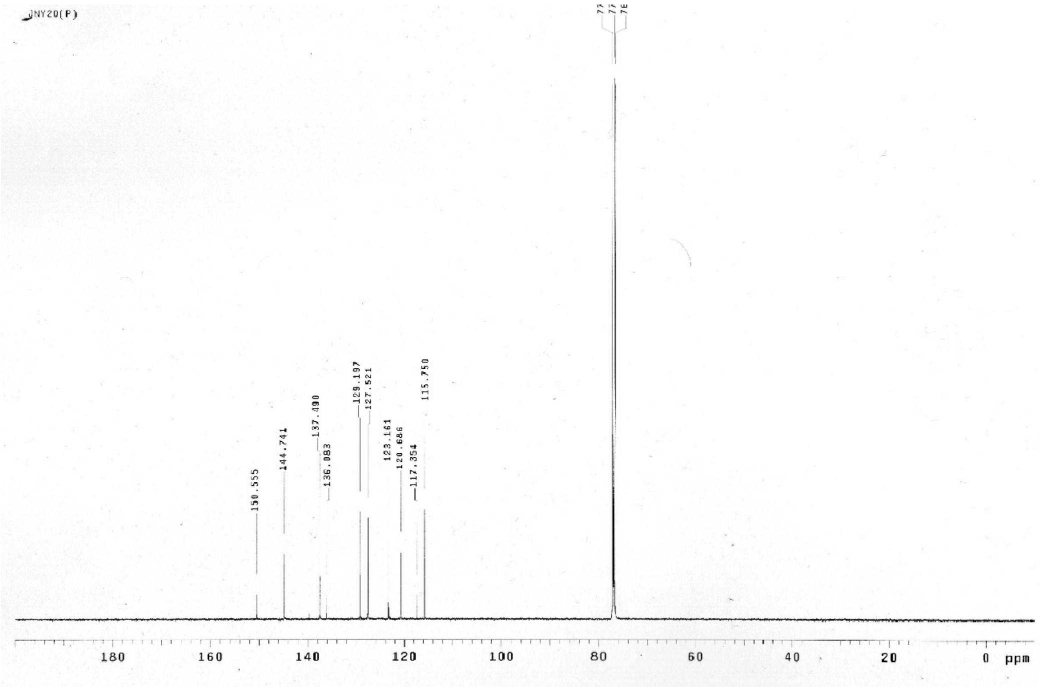

Figure S20: ^{13}C NMR of **7**

3-Chloro-N-(2-nitrophenyl)pyridin-2-amine (8)

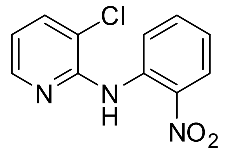

IR (KBr) ν 3263 cm^{-1}. ^{1}H NMR (500 MHz, DMSO-d$_6$) δ 6.89 (dd, J = 5.0, 7.5 Hz, 1H), 7.01 (ddd, J = 1.5, 7.0, 8.5 Hz, 1H), 7.60 (ddd, J = 1.5, 7.0, 8.5 Hz, 1H), 7.69 (dd, J = 1.5, 7.5 Hz, 1H), 8.21 (dd, J = 1.5, 5.0 Hz, 1H), 8.24 (dd, J = 1.5, 8.5 Hz, 1H), 9.06 (dd, J = 1.5, 8.5 Hz, 1H), 10.79 (s, 1H, NH). ^{13}C NMR (125 MHz, DMSO-d$_6$) δ 117.5, 118.9, 120.3, 120.5, 126.0, 135.3, 135.5, 137.4, 137.6, 145.3, 150.1. EI-MS m/z: 251 (M+2), 249 (M+), 218, 203, 168, 140, 114, 76, 63, 50. EI-HRMS (m/z) calcd for $C_{11}H_8ClN_3O_2$: 249.0305; found: 249.0302.

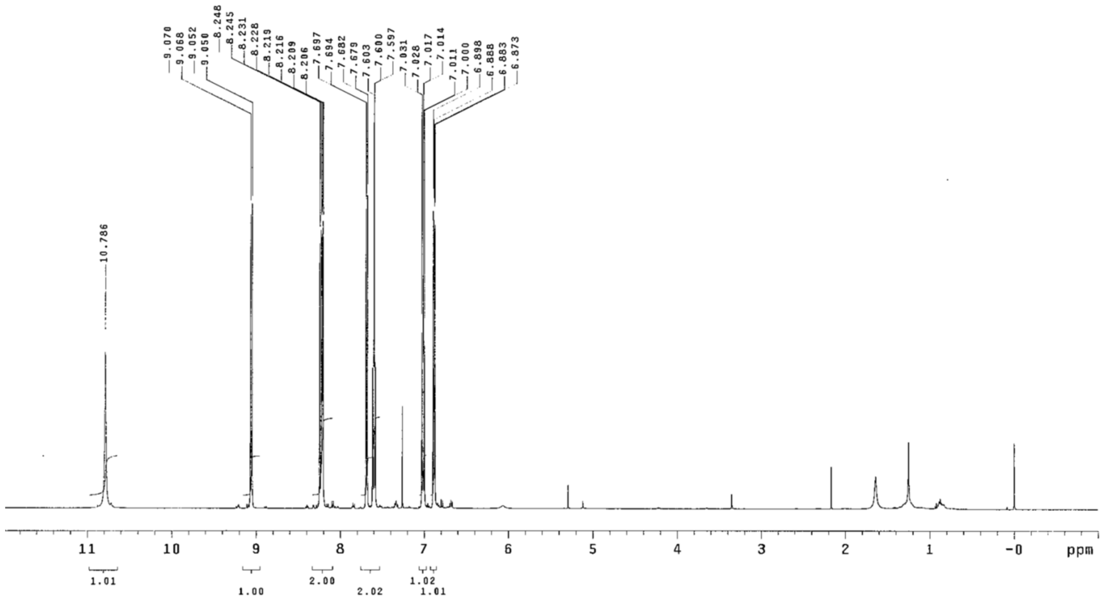

Figure S21: ^{1}H NMR of **8**

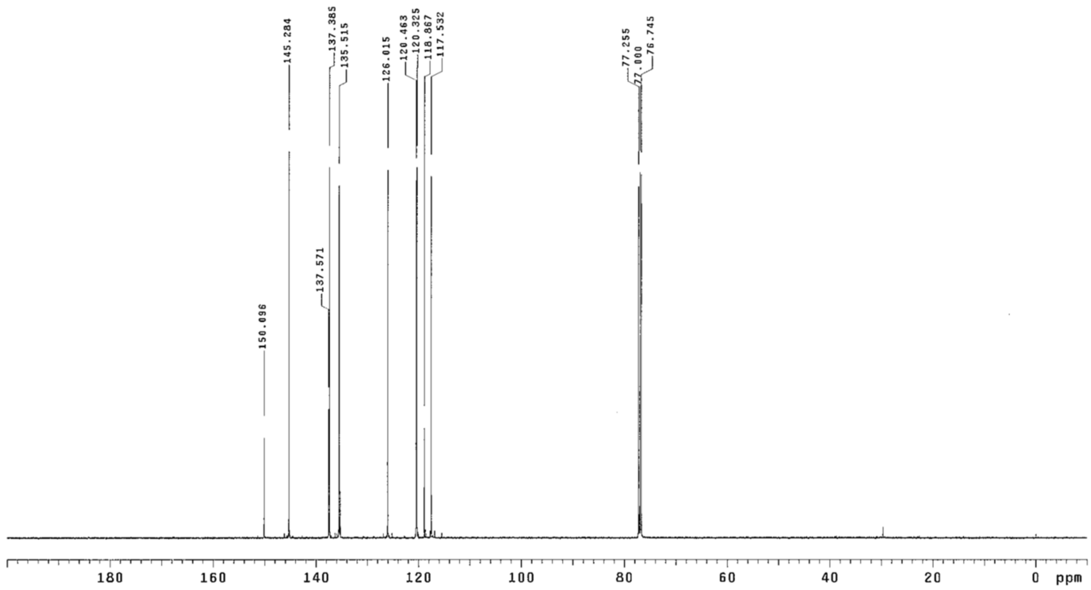

Figure S22: ^{13}C NMR of **8**

3-Chloro-N-(4-chloro-2-methylphenyl)pyridin-2-amine (9)

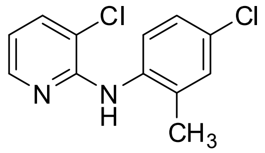

IR (KBr) ν 3426 cm^{-1}. ^{1}H NMR (400 MHz, $CDCl_3$) δ 2.29 (s, 3H, CH_3), 6.71 (dd, J = 4.8, 7.6 Hz, 1H), 6.84 (br s, 1H, NH), 7.19–7.21 (m, 2H), 7.60 (dd, J = 1.6, 7.6 Hz, 1H), 7.94 (d, J = 9.2 Hz, 1H), 8.10 (dd, J = 1.6, 4.8 Hz, 1H). ^{13}C NMR (100 MHz, $CDCl_3$) δ 17.8, 115.1, 116.4, 123.2, 126.6, 128.6, 130.2, 131.1, 136.2, 137.0, 145.5, 151.3. EI-MS m/z: 256 (M+4), 254 (M+2), 252 (M+), 237, 216, 202, 181, 167, 154, 140, 125, 112, 108, 90, 77, 63, 51. EI-HRMS (m/z) calcd for $C_{12}H_{10}Cl_2N_2$: 252.0221; found: 252.0218.

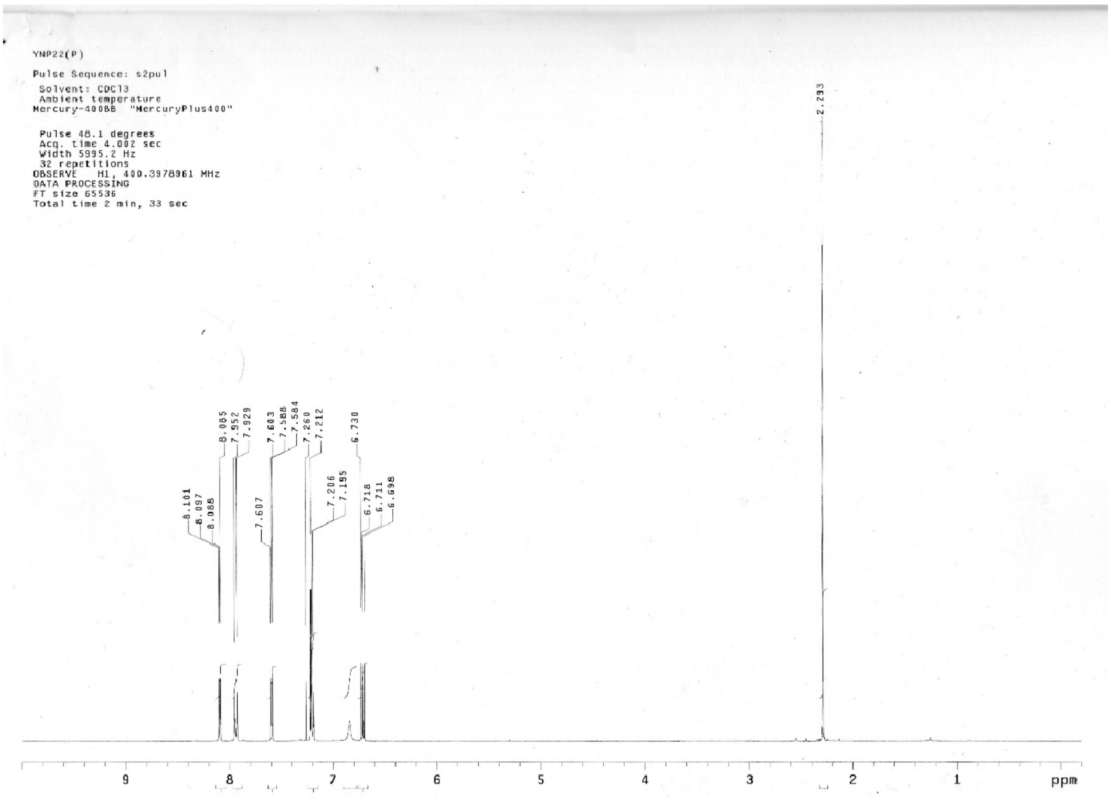

Figure S23: ^{1}H NMR of **9**

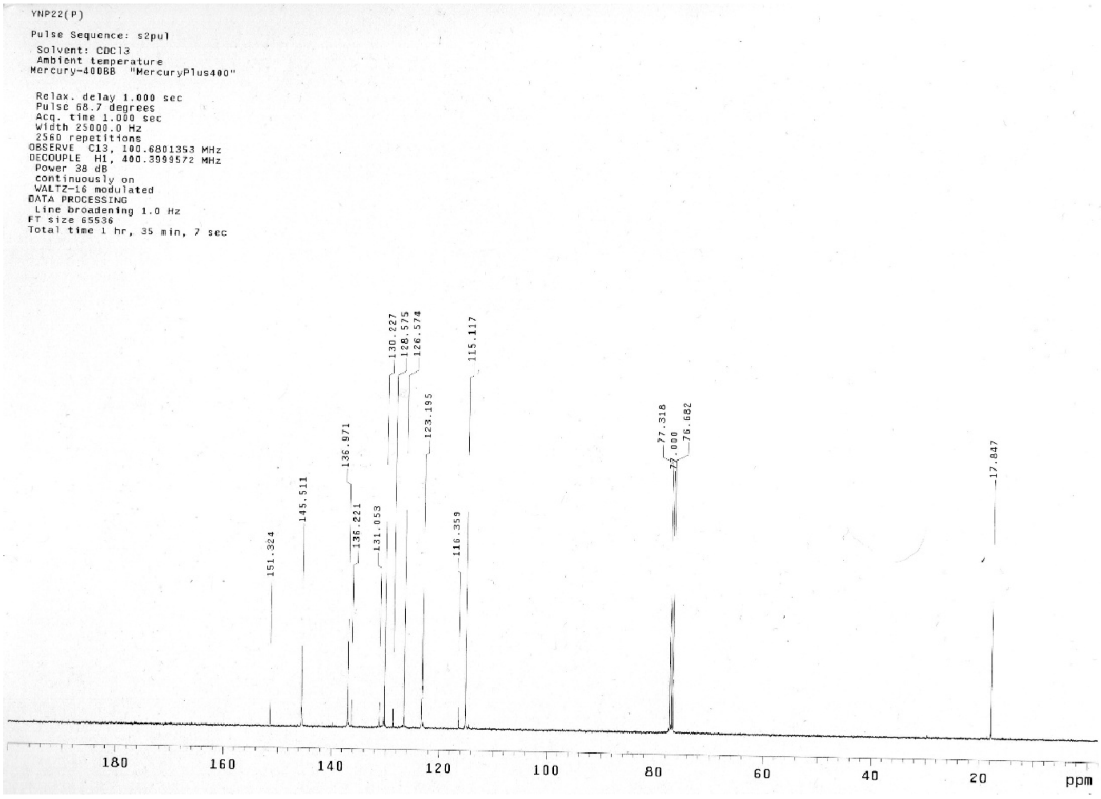

Figure S24: ^{13}C NMR of **9**

3-Chloro-N-(2,4-dimethylphenyl)pyridin-2-amine (10)

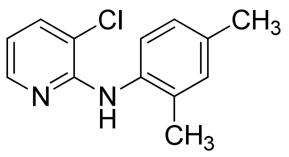

IR (KBr) ν 3426 cm^{-1}. ^1H NMR (500 MHz, $CDCl_3$) δ 2.28 (s, 3H, CH_3), 2.33 (s, 3H, CH_3), 6.66 (dd, J = 5.0, 8.0 Hz, 1H), 6.74 (br s, 1H, NH), 7.07 (d, J = 8.0 Hz, 1H), 7.08 (s, 1H), 7.57 (dd, J = 1.5, 8.0 Hz, 1H), 7.70 (d, J = 8.0 Hz, 1H), 8.08 (dd, J = 1.5, 5.0 Hz, 1H). ^{13}C NMR (125 MHz, $CDCl_3$) δ 17.9, 20.9, 114.3, 116.0, 123.3, 127.2, 130.5, 131.3, 134.0, 134.9, 136.6. EI-MS m/z: 234 (M+2), 232 (M+), 217, 196, 181, 154, 127, 115, 91, 77, 51. EI-HRMS (m/z) calcd for $C_{13}H_{13}ClN_2$: 232.0767; found: 232.0770.

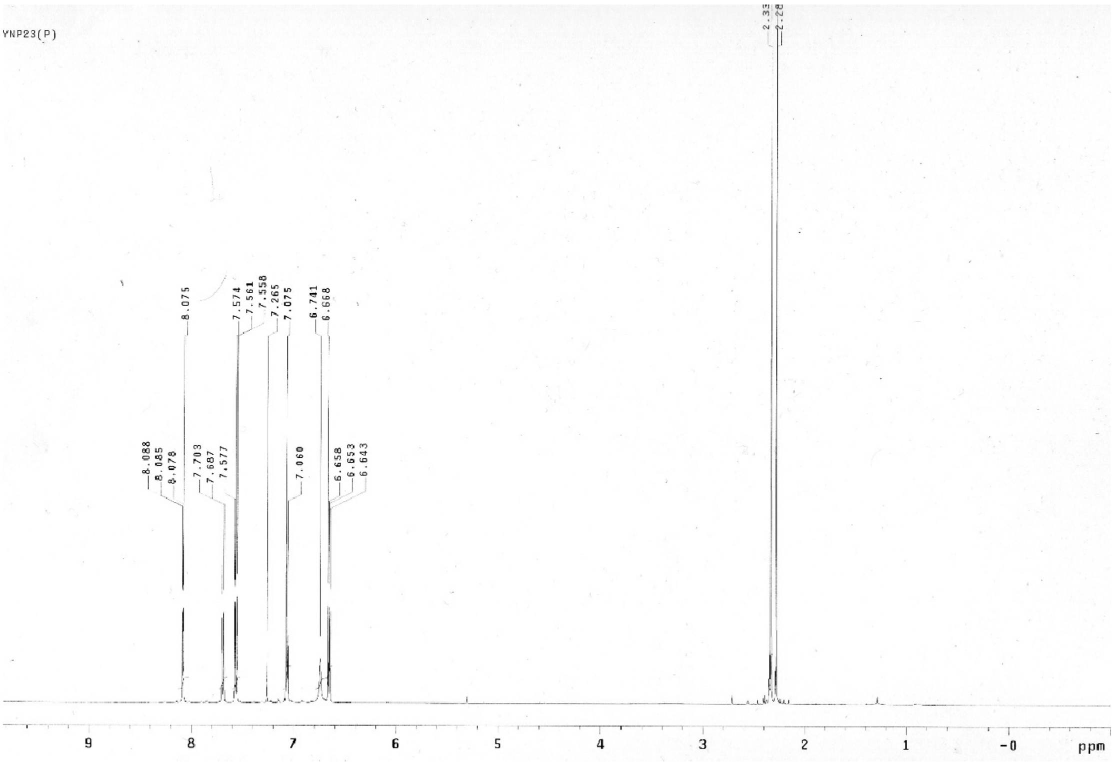

Figure S25: ^1H NMR of **10**

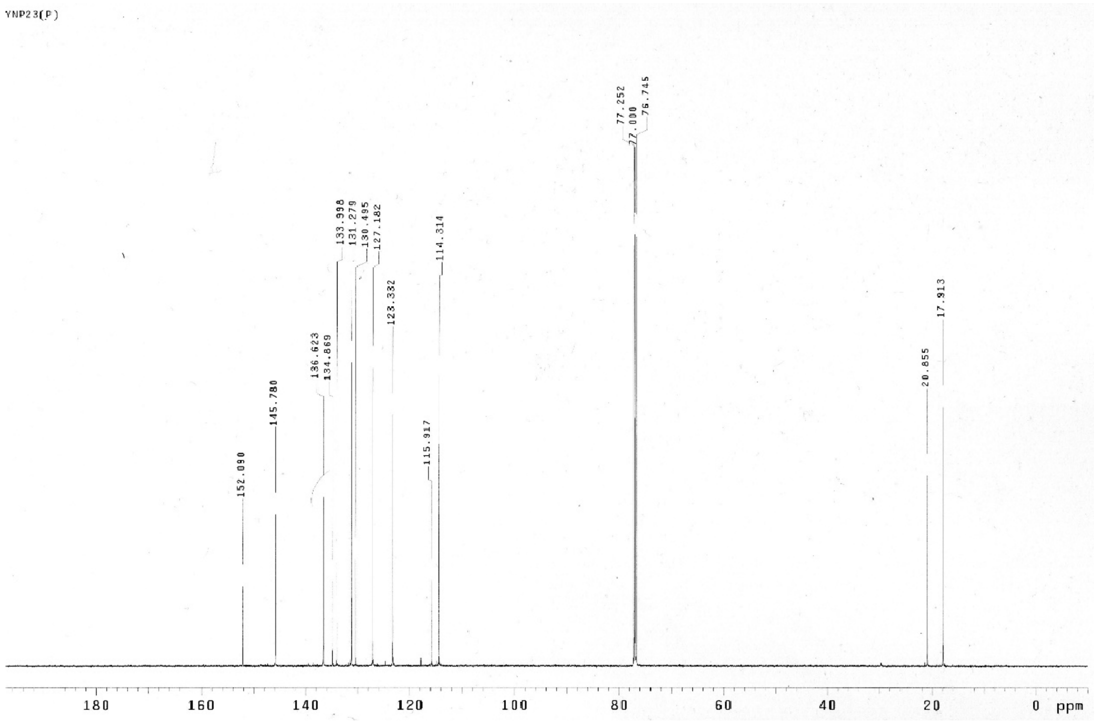

Figure S26: ^{13}C NMR of **10**

3-Chloro-N-(2-chloro-4-methylphenyl)pyridin-2-amine (11)

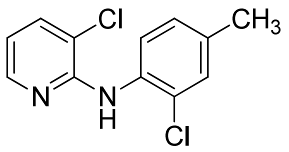

IR (KBr) ν 3397 cm^{-1}. ^1H NMR (400 MHz, $CDCl_3$) δ 2.30 (s, 3H, CH_3), 6.72 (dd, J = 4.8, 7.6 Hz, 1H), 7.08 (dd, J = 1.6, 8.4 Hz, 1H), 7.21 (d, J = 1.6 Hz, 1H), 7.54 (br s, 1H, NH), 7.58 (dd, J = 1.6, 7.6 Hz, 1H), 8.13 (dd, J = 1.6, 4.8 Hz, 1H), 8.41 (d, J = 8.4 Hz, 1H). ^{13}C NMR (100 MHz, $CDCl_3$) δ 20.5, 115.5, 116.8, 120.4, 122.8, 128.0, 129.4, 132.6, 133.8, 136.7, 145.6, 150.9. EI-MS m/z: 256, 254, 252, 217, 202, 181, 167, 154, 127, 108, 90, 76, 63, 51. EI-HRMS (m/z) calcd for $C_{12}H_{10}Cl_2N_2$: 252.0221; found: 252.0220.

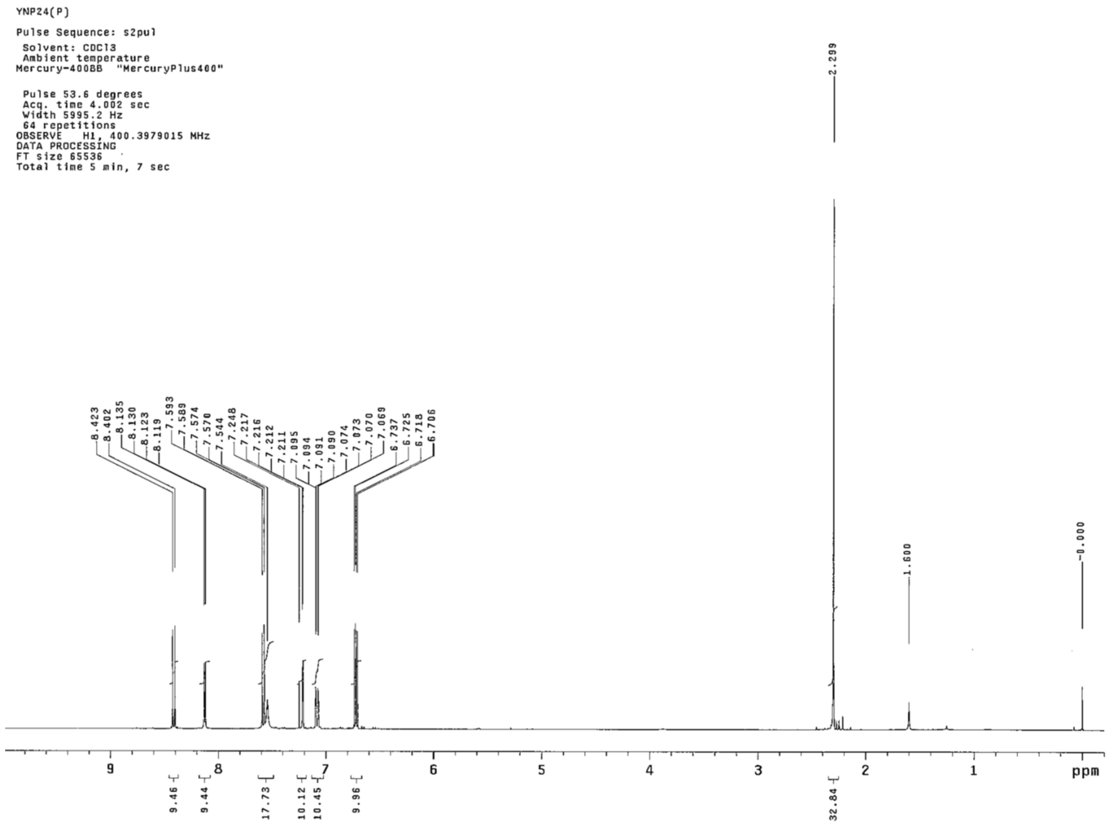

Figure S27: ^{1}H NMR of **11**

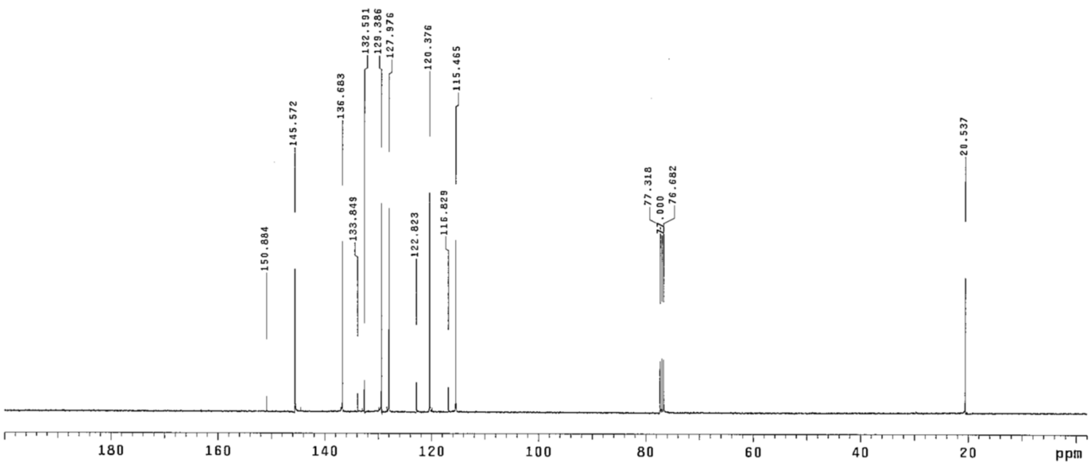

Figure S28: ^{13}C NMR of **11**

3-Chloro-N-(2,6-dimethylphenyl)pyridin-2-amine (12)

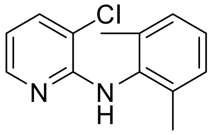

IR (KBr) ν 3378 cm^{-1}. ^{1}H NMR (400 MHz, $CDCl_3$) δ 2.21 (s, 6H, CH_3 ×2), 6.29 (br s, 1H, NH), 6.57 (dd, J = 4.8, 7.6 Hz, 1H), 7.13 (br s, 3H), 7.53 (dd, J = 1.6, 7.6 Hz, 1H), 7.97 (dd, J = 1.6, 4.8 Hz, 1H). ^{13}C NMR (100 MHz, $CDCl_3$) δ 18.5, 113.8, 115.2, 126.8, 128.2, 136.1, 136.2, 136.4, 146.5, 152.8. EI-MS m/z: 234 (M+2), 232 (M+), 217, 196, 181, 167, 154, 127, 115, 91, 77, 51. EI-HRMS (m/z) calcd for $C_{13}H_{13}ClN_2$: 232.0767; found: 232.0768.

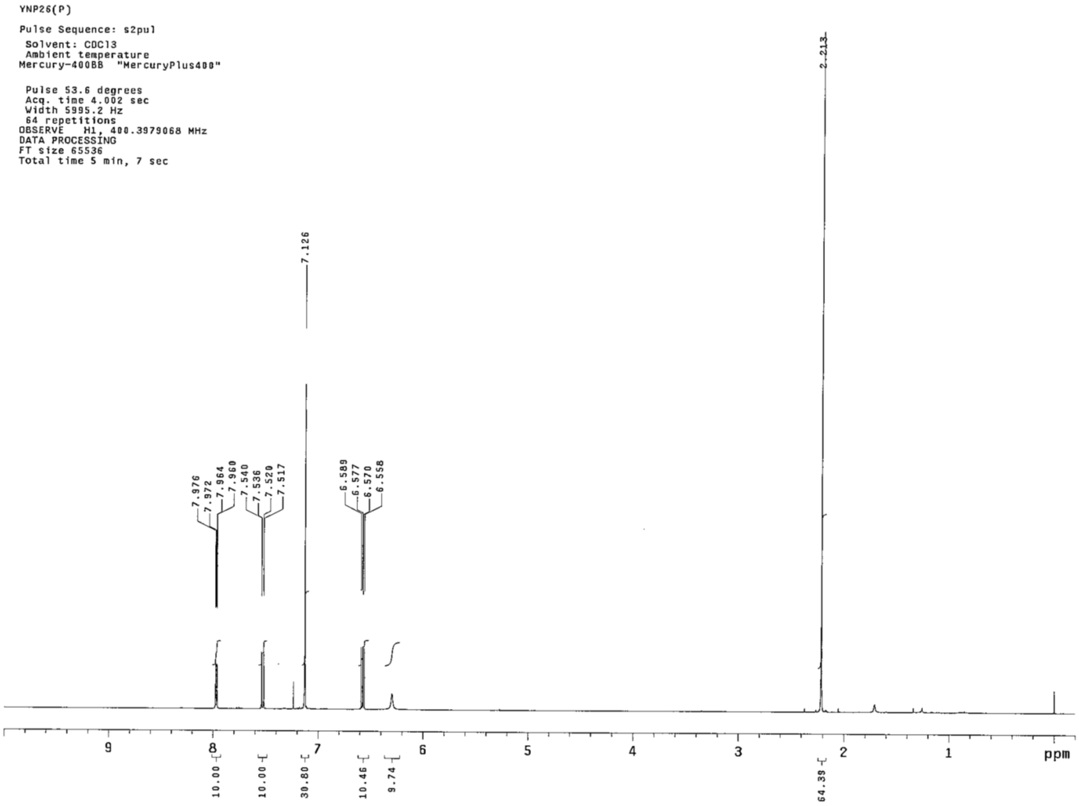

Figure S29: ^{1}H NMR of **12**

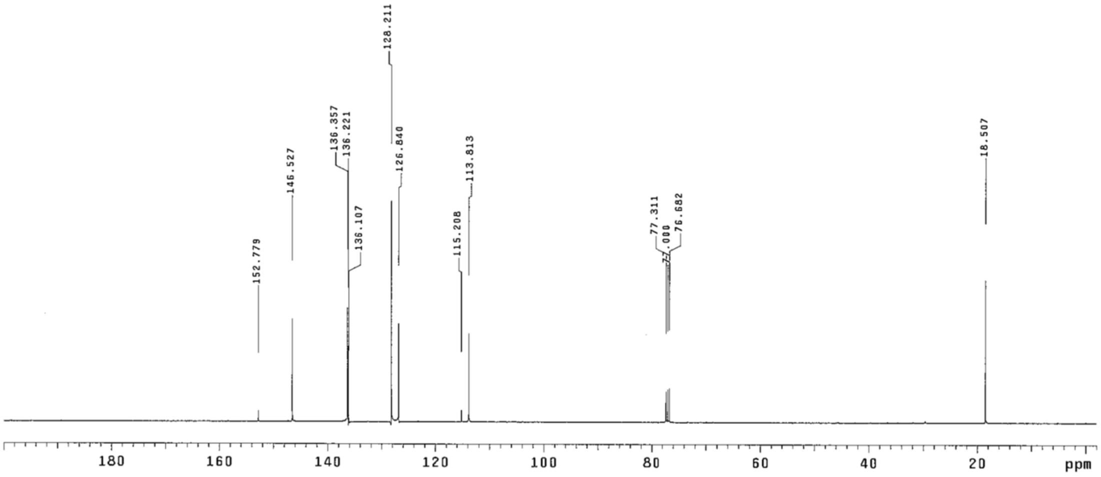

Figure S30: ^{13}C NMR of **12**

3-Chloro-N-(m-tolyl)pyridin-2-amine (13)

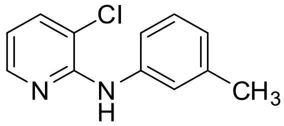

IR (KBr) ν 3416 cm^{-1}. ^{1}H NMR (500 MHz, $CDCl_3$) δ 2.39 (s, 3H, CH_3), 6.71 (dd, J = 5.0, 7.5 Hz, 1H), 6.90 (d, J = 7.5 Hz, 1H), 6.98 (br s, 1H, NH), 7.25 (dd, J = 7.5, 8.0 Hz, 1H), 7.43 (s, 1H), 7.46 (d, J = 8.0 Hz, 1H), 7.58 (dd, J = 1.5, 7.5 Hz, 1H), 8.14 (dd, J = 1.5, 5.0 Hz, 1H). ^{13}C NMR (125 MHz, $CDCl_3$) δ 21.5, 114.9, 116.0, 117.2, 120.7, 123.8, 128.8, 136.7, 138.7, 139.4, 145.6, 151.3. EI-MS m/z: 220 (M+2), 218 (M+), 202, 181, 168, 140, 127, 108, 90, 77, 76, 63. EI-HRMS (m/z) calcd for $C_{12}H_{11}ClN_2$: 218.0611; found: 218.0614.

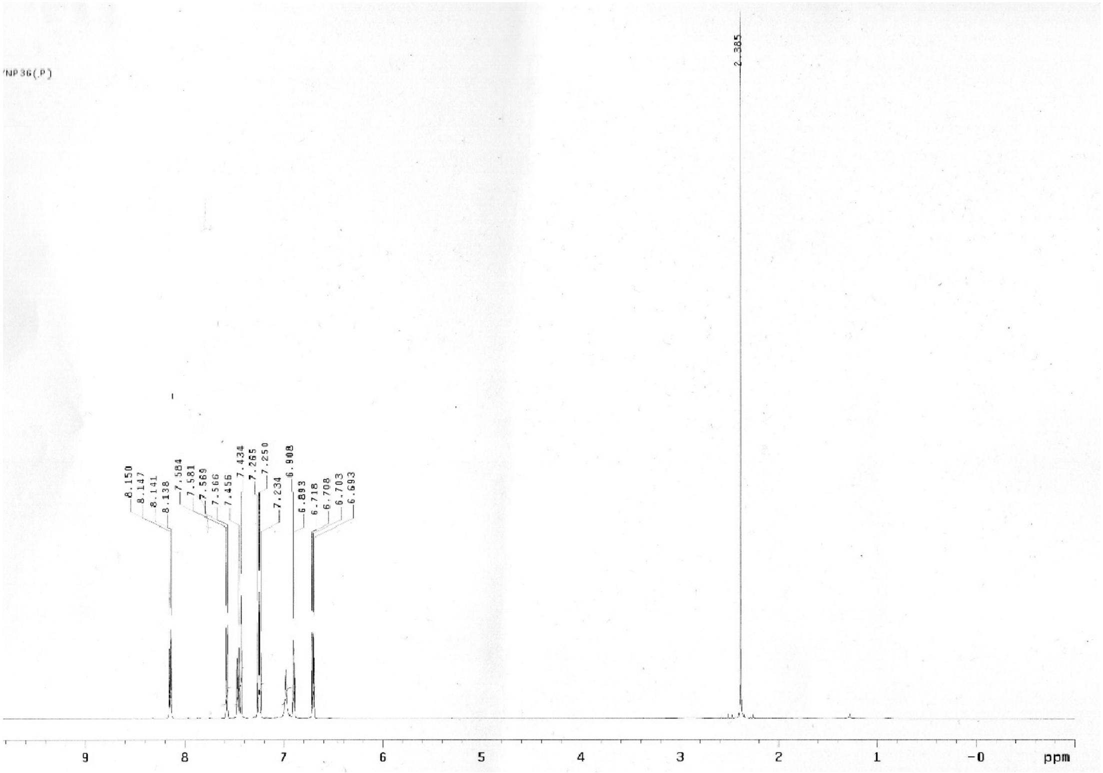

Figure S31: ^{1}H NMR of **13**

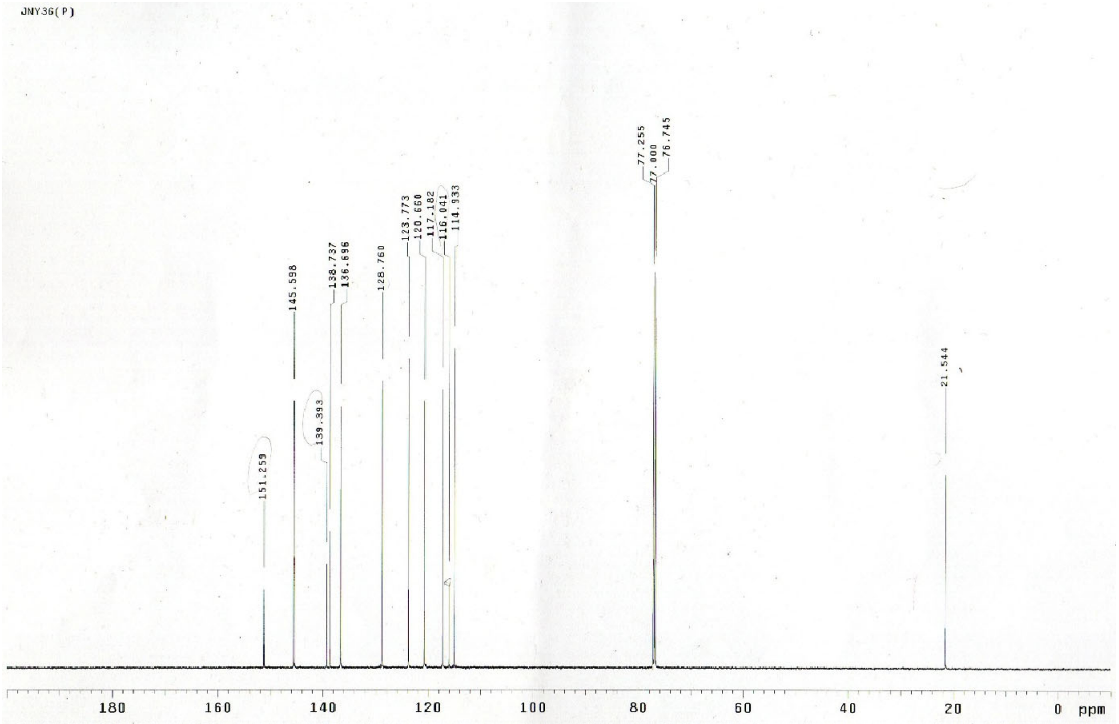

Figure S32: ^{13}C NMR of **13**

N-(3-(Benzyloxy)phenyl)-3-chloropyridin-2-amine (14)

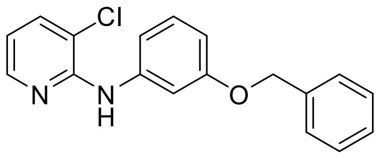

IR (KBr) ν 3407 cm^{-1}. ^1H NMR (400 MHz, $CDCl_3$) δ 5.11 (s, 2H, CH_2), 6.71 (dd, J = 0.8, 8.0 Hz, 1H), 6.72 (dd, J = 4.8, 8.0 Hz, 1H), 7.05 (br s, 1H, NH), 7.14 (ddd, J = 0.8, 2.0, 8.0 Hz, 1H), 7.26 (t, J = 8.0 Hz, 1H), 7.35 (tt, J = 1.6, 7.2 Hz, 1H), 7.42 (t, J = 7.2 Hz, 2H), 7.50 (dd, J = 1.6, 7.2 Hz, 2H), 7.57 (d, J = 2.0 Hz, 1H), 7.59 (dd, J = 1.6, 8.0 Hz, 1H), 8.16 (dd, J = 1.6, 4.8 Hz, 1H). ^{13}C NMR (100 MHz, $CDCl_3$) δ 69.9, 106.6, 109.0, 112.4, 115.1, 116.1, 127.5, 127.8, 128.5, 129.5, 136.7, 137.0, 140.8. EI-MS m/z: 312 (M+2), 310 (M+), 275, 219, 205, 197, 191, 155, 128, 112, 91, 76, 65. EI-HRMS (m/z) calcd for $C_{18}H_{15}ClN_2O$: 310.0873; found: 310.0870.

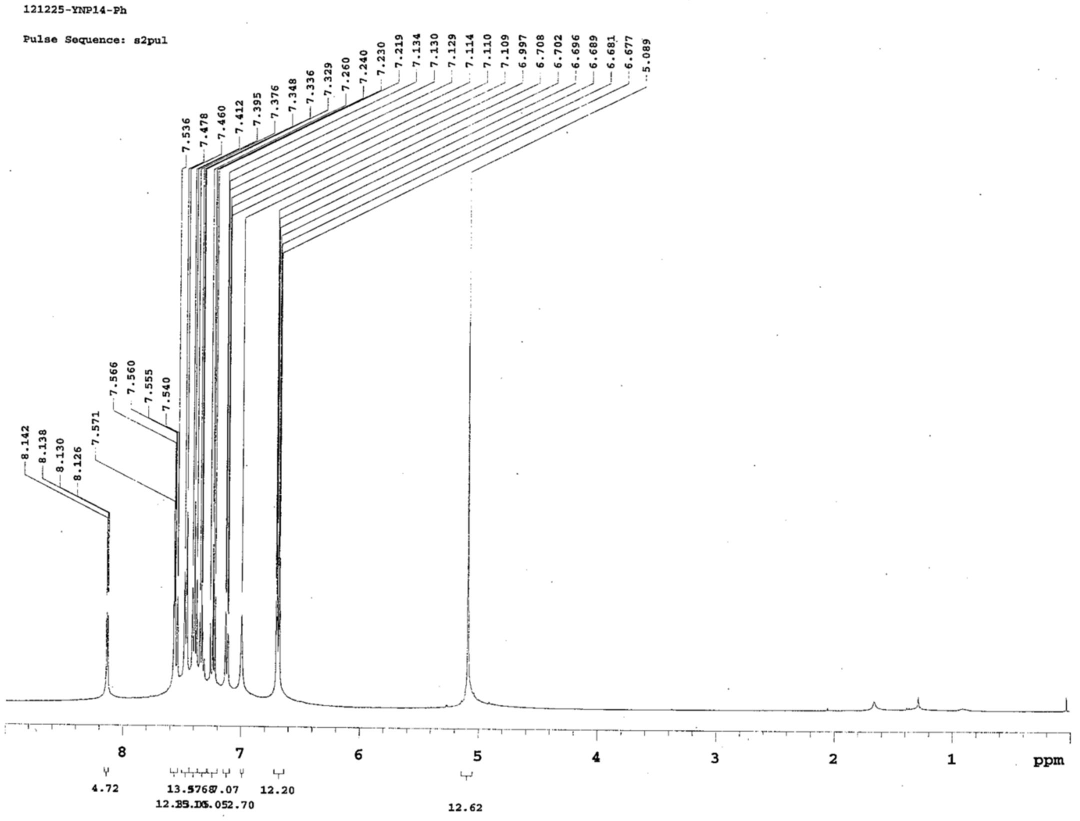

Figure S33: ^1H NMR of **14**

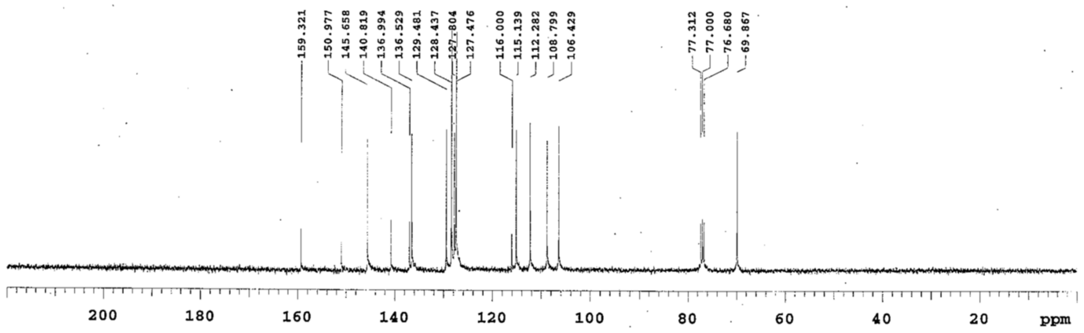

Figure S34: ^{13}C NMR of **14**

3-Chloro-N-(3-nitrophenyl)pyridin-2-amine (15)

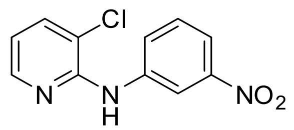

IR (KBr) ν 3378 cm^{-1}. ^{1}H NMR (500 MHz, DMSO-d_6) δ 6.94 (dd, J = 5.0, 7.5 Hz, 1H), 7.55 (t, J = 8.0 Hz, 1H), 7.80 (dd, J = 2.0, 8.0 Hz, 1H), 7.86 (dd, J = 1.5, 7.5 Hz, 1H), 8.12 (dd, J = 2.0, 8.0 Hz, 1H), 8.18 (dd, J = 1.5, 5.0 Hz, 1H), 8.71 (t, J = 2.0 Hz, 1H), 8.92 (br s, 1H, NH). ^{13}C NMR (125 MHz, DMSO-d_6) δ 113.9, 116.3, 116.6, 117.2, 126.2, 129.8, 138.2, 142.0, 145.8, 148.1, 150.8. EI-MS m/z: 251 (M+2), 249 (M+), 232, 218, 202, 168, 140, 114, 84, 76, 63, 50. EI-HRMS (m/z) calcd for $C_{11}H_8ClN_3O_2$: 249.0305; found: 249.0308.

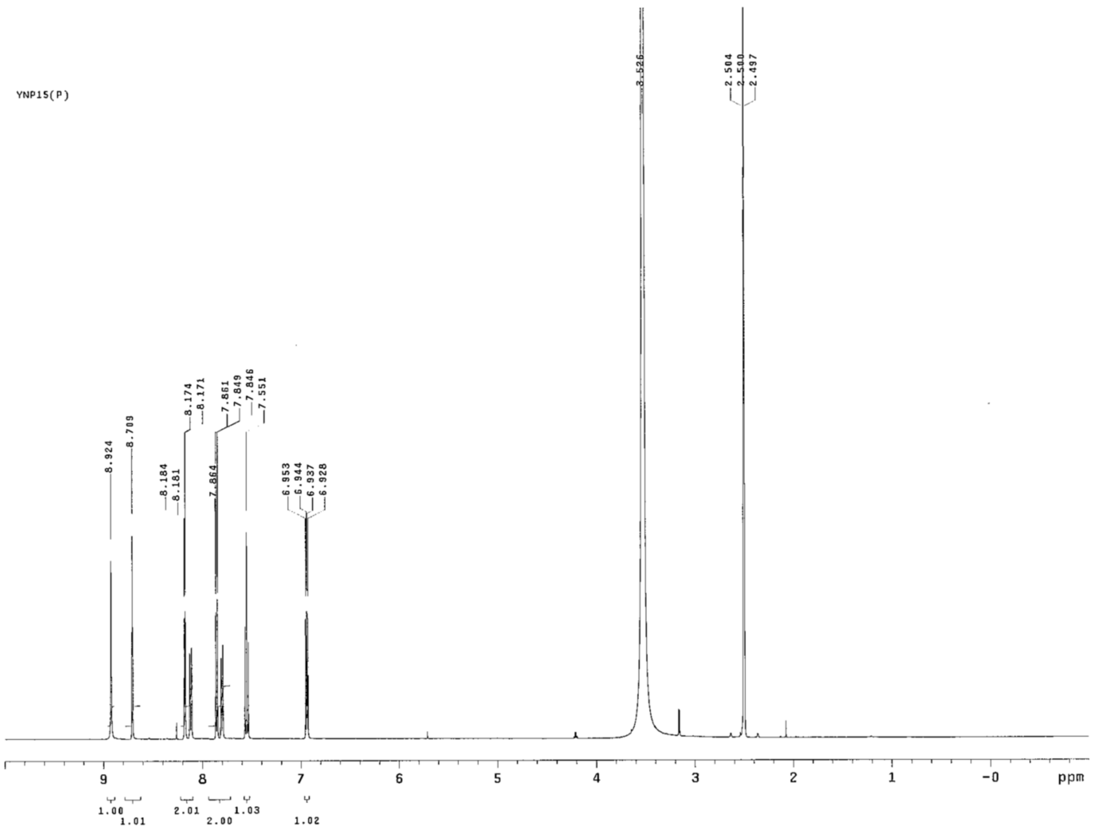

Figure S35: ^{1}H NMR of **15**

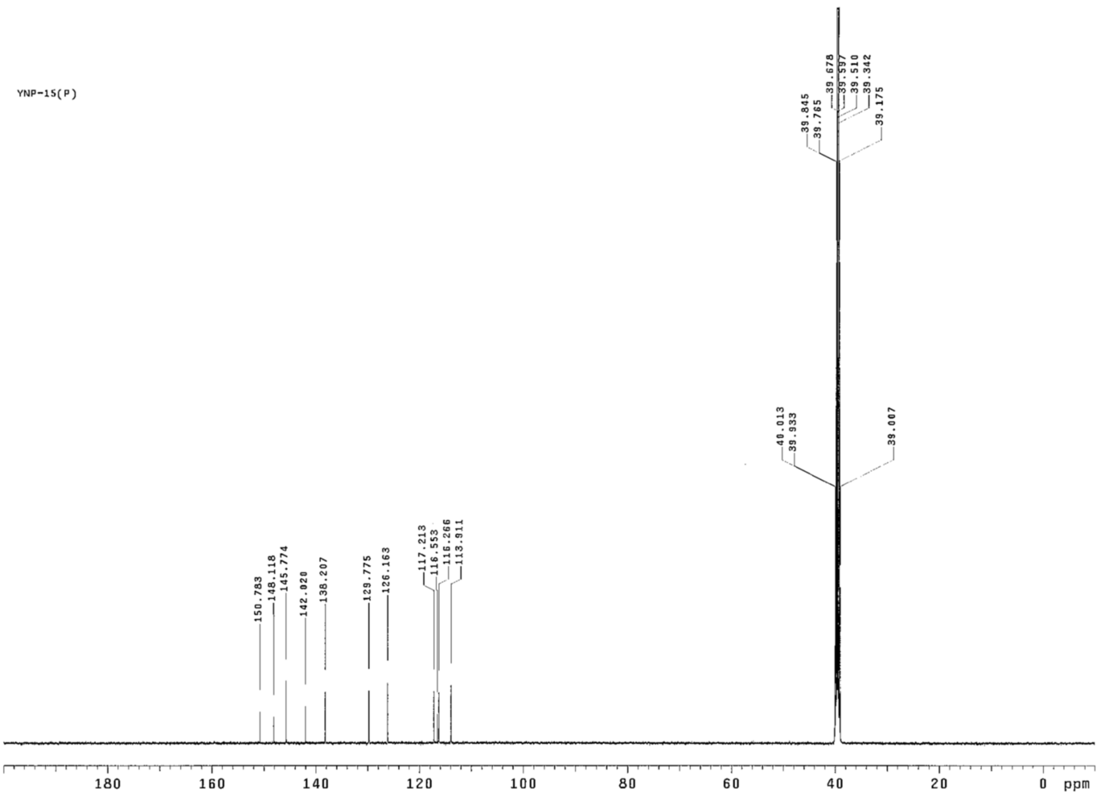

Figure S36: ^{13}C NMR of **15**

3-Chloro-N-(3,5-dimethoxyphenyl)pyridin-2-amine (16)

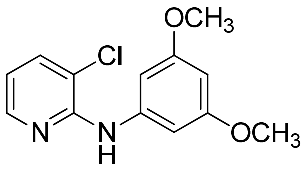

IR (KBr) ν 3407 cm^{-1}. ^{1}H NMR (400 MHz, $CDCl_3$) δ 3.78 (s, 6H, OCH_3), 6.18 (t, J = 2.4 Hz, 1H), 6.68 (dd, J = 5.2, 8.0 Hz, 1H), 6.88 (d, J = 2.4 Hz, 2H), 6.98 (br s, 1H, NH), 7.54 (dd, J = 1.6, 8.0 Hz, 1H), 8.12 (dd, J = 1.6, 5.2 Hz, 1H). ^{13}C NMR (100 MHz, $CDCl_3$) δ 55.3, 94.9, 98.1, 115.2, 116.1, 136.7, 141.2, 145.6, 151.0, 161.0. EI-MS m/z: 266 (M+2), 264 (M+), 248, 229, 214, 206, 185, 155, 112, 76, 51. EI-HRMS (m/z) calcd for $C_{13}H_{13}ClN_2O_2$: 264.0666; found: 264.0666.

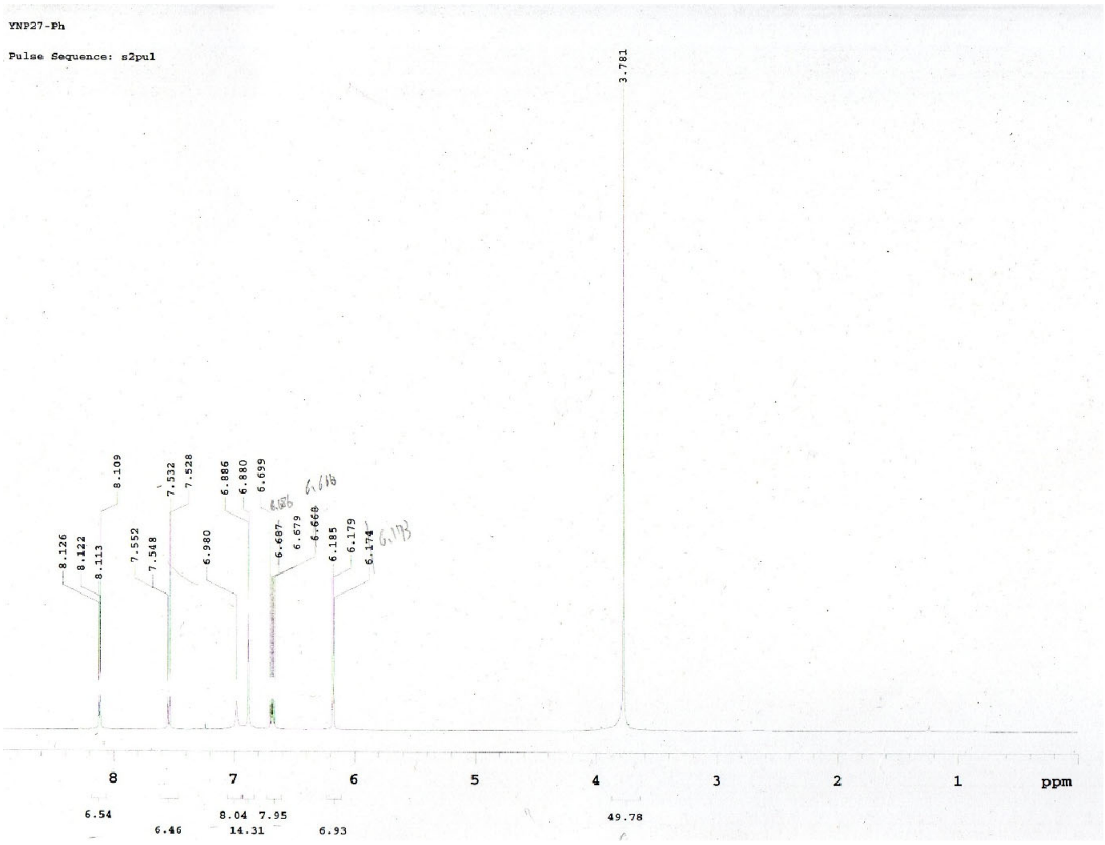

Figure S37: ^{1}H NMR of **16**

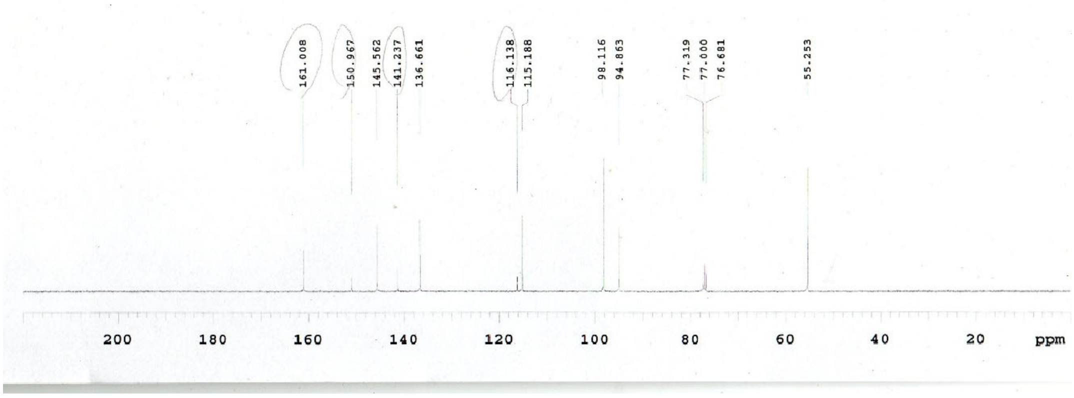

Figure S38: ^{13}C NMR of **16**

3-Chloro-N-(p-tolyl)pyridin-2-amine (17)

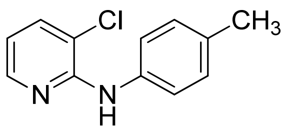

IR (KBr) ν 3407 cm^{-1}. ^{1}H NMR (500 MHz, $CDCl_3$) δ 2.35 (s, 3H, CH_3), 6.68 (dd, J = 5.0, 8.0 Hz, 1H), 6.94 (br s, 1H, NH), 7.17 (d, J = 8.0 Hz, 2H), 7.50 (d, J = 8.0 Hz, 2H), 7.56 (dd, J = 1.5, 8.0 Hz, 1H), 8.12 (dd, J = 1.5, 5.0 Hz, 1H). ^{13}C NMR (125 MHz, $CDCl_3$) δ 20.8, 114.7, 115.8, 120.6, 129.4, 132.6, 136.6, 136.8, 145.6, 151.5. EI-MS m/z: 220 (M+2), 218 (M+), 202, 181, 168, 140, 90, 77. EI-HRMS (m/z) calcd for $C_{12}H_{11}ClN_2$: 218.0611; found: 218.0610.

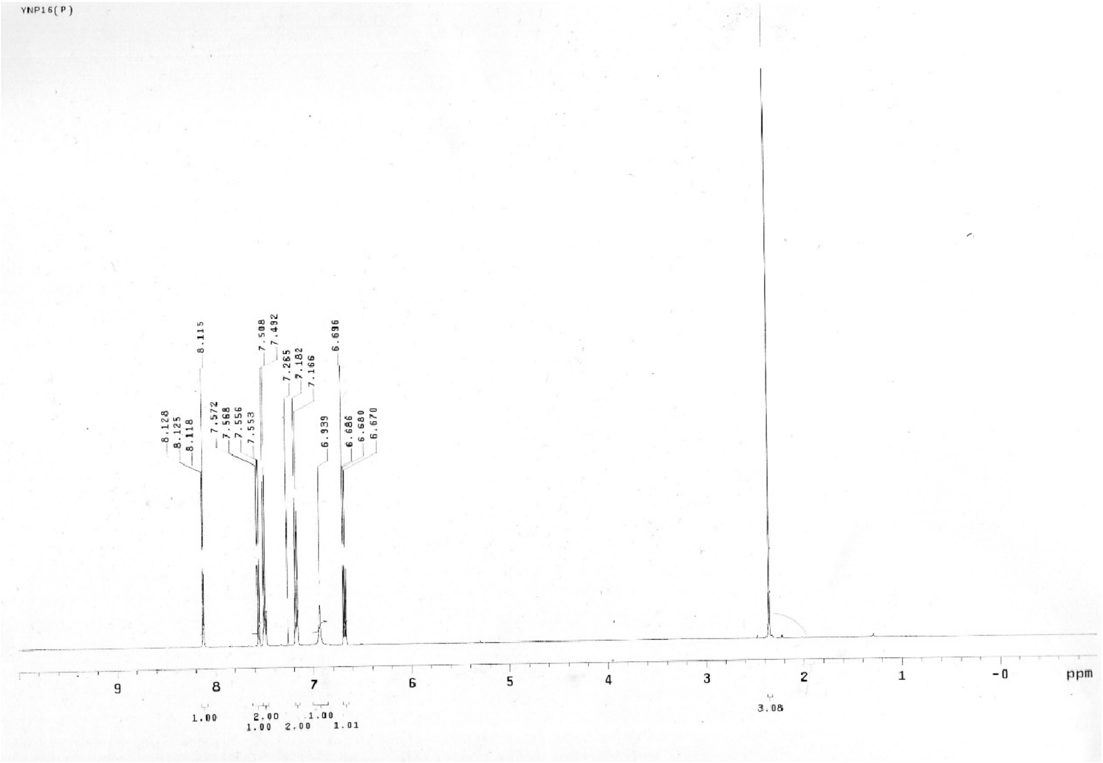

Figure S39: ^{1}H NMR of **17**

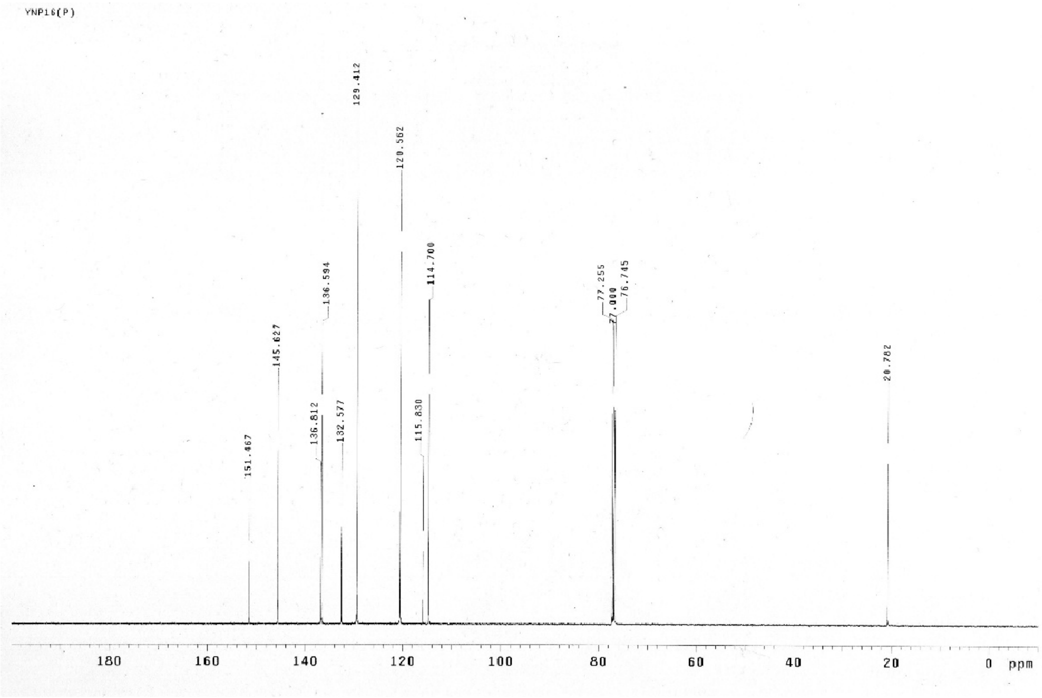

Figure S40: ^{13}C NMR of **17**

3-Chloro-N-(4-methoxyphenyl)pyridin-2-amine (18)

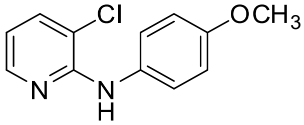

IR (KBr) ν 3416 cm^{-1}. ^{1}H NMR (400 MHz, $CDCl_3$) δ 3.81 (s, 3H, OCH_3), 6.66 (dd, J = 5.2, 8.0 Hz, 1H), 6.89 (br s, 1H, NH), 6.91 (d, J = 8.8 Hz, 2H), 7.46 (d, J = 8.8 Hz, 2H), 7.56 (dd, J = 1.6, 8.0 Hz, 1H), 8.08 (dd, J = 1.6, 5.2 Hz, 1H). ^{13}C NMR (100 MHz, $CDCl_3$) δ 55.5, 114.3, 114.5, 115.8, 123.0, 132.2, 136.9, 145.4, 151.7, 156.0. EI-MS m/z: 236 (M+2), 234 (M+), 219, 191, 155, 128, 112, 101, 76, 63, 51. EI-HRMS (m/z) calcd for $C_{12}H_{11}ClN_2O$: 234.0560; found: 234.0561.

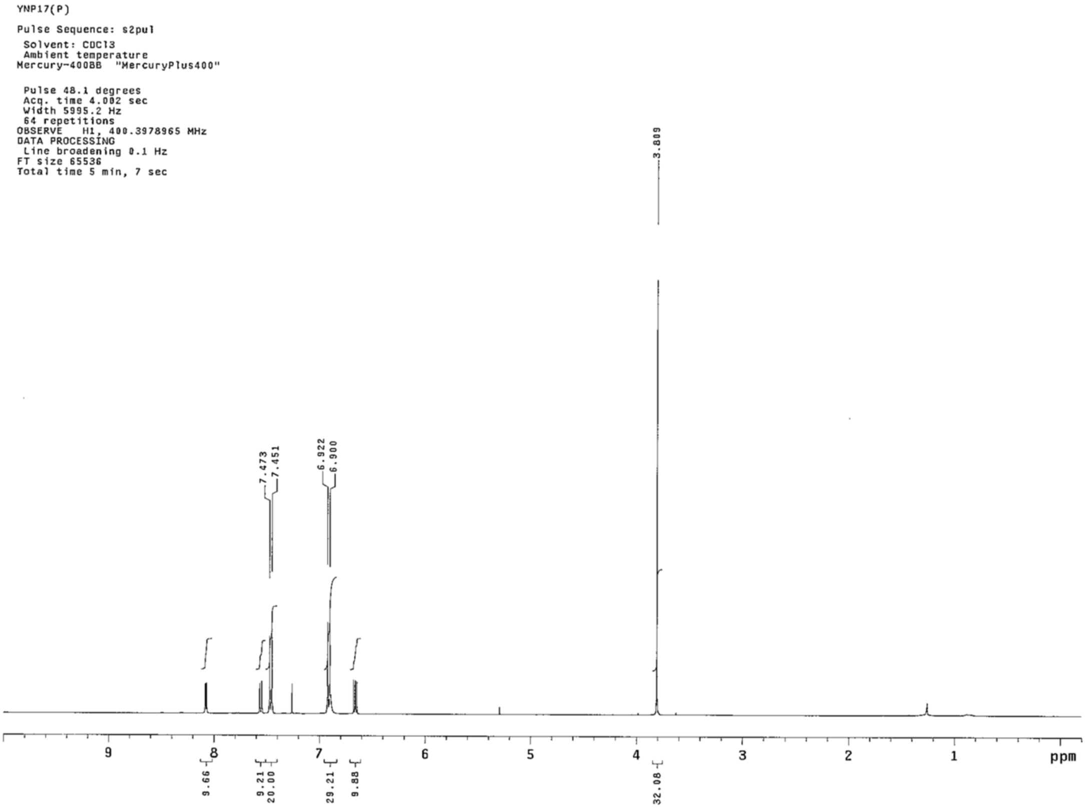

Figure S41: ^{1}H NMR of **18**

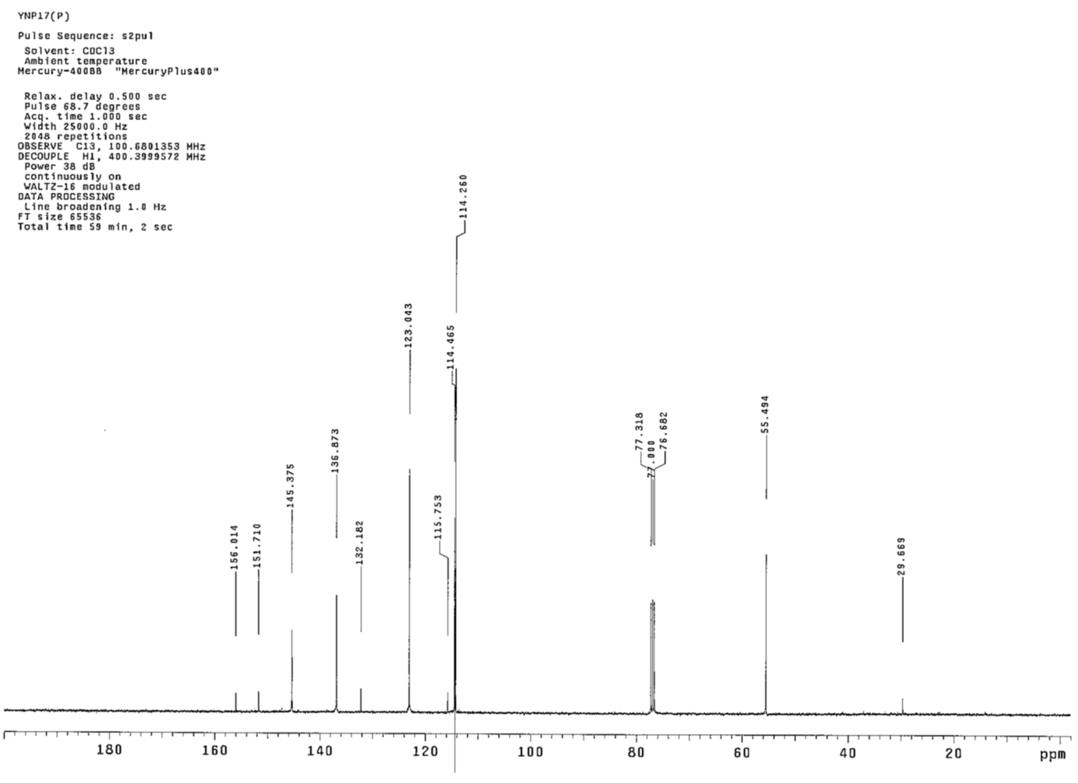

Figure S42: ^{13}C NMR of **18**

3-Chloro-N-(4-chlorophenyl)pyridin-2-amine (19)

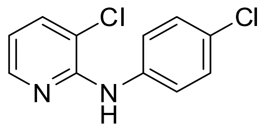

IR (KBr) ν 3407 cm^{-1}. ^{1}H NMR (500 MHz, $CDCl_3$) δ 6.73 (dd, J = 5.0, 7.5 Hz, 1H), 6.97 (br s, 1H, NH), 7.30 (d, J = 9.0 Hz, 2H), 7.58 (dd, J = 1.5, 7.5 Hz, 1H), 7.60 (d, J = 9.0 Hz, 2H), 8.13 (dd, J = 1.5, 5.0 Hz, 1H). ^{13}C NMR (125 MHz, $CDCl_3$) δ 115.5, 116.1, 121.0, 127.4, 128.8, 136.7, 138.2, 145.6, 150.9. EI-MS m/z: 242 (M+4), 240 (M+2), 238 (M+), 202, 168, 140, 114, 101, 84, 75, 63, 50. EI-HRMS (m/z) calcd for $C_{11}H_8Cl_2N_2$: 238.0065; found: 238.0062.

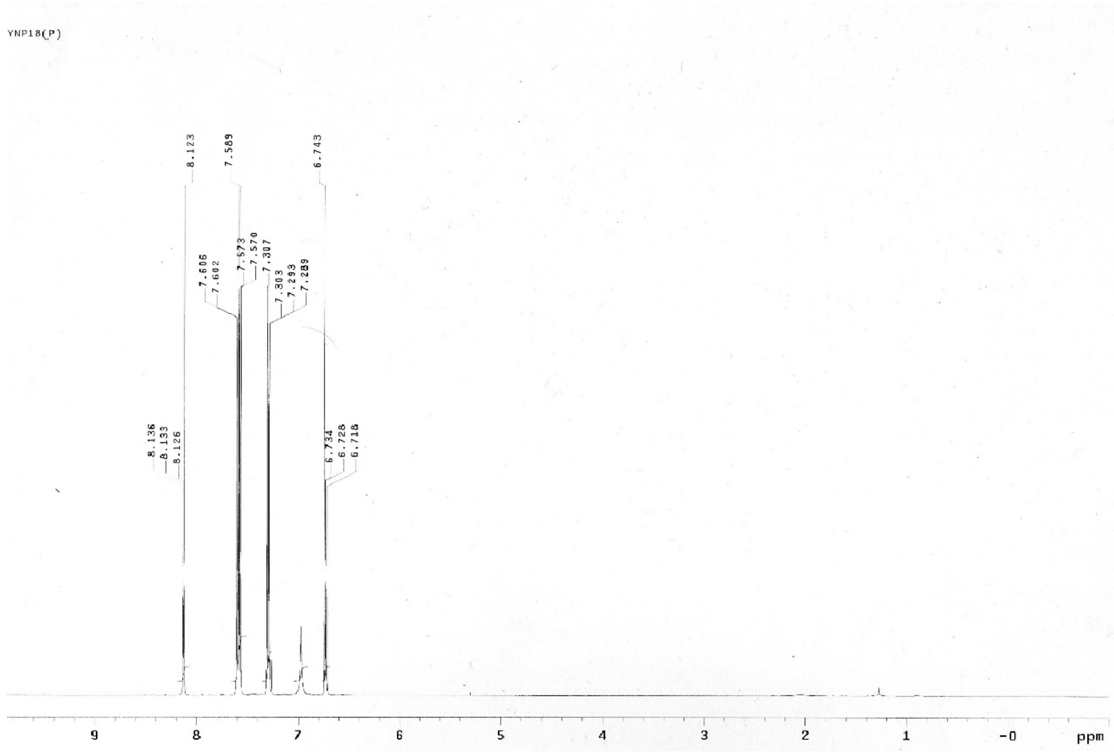

Figure S43: ^{1}H NMR of **19**

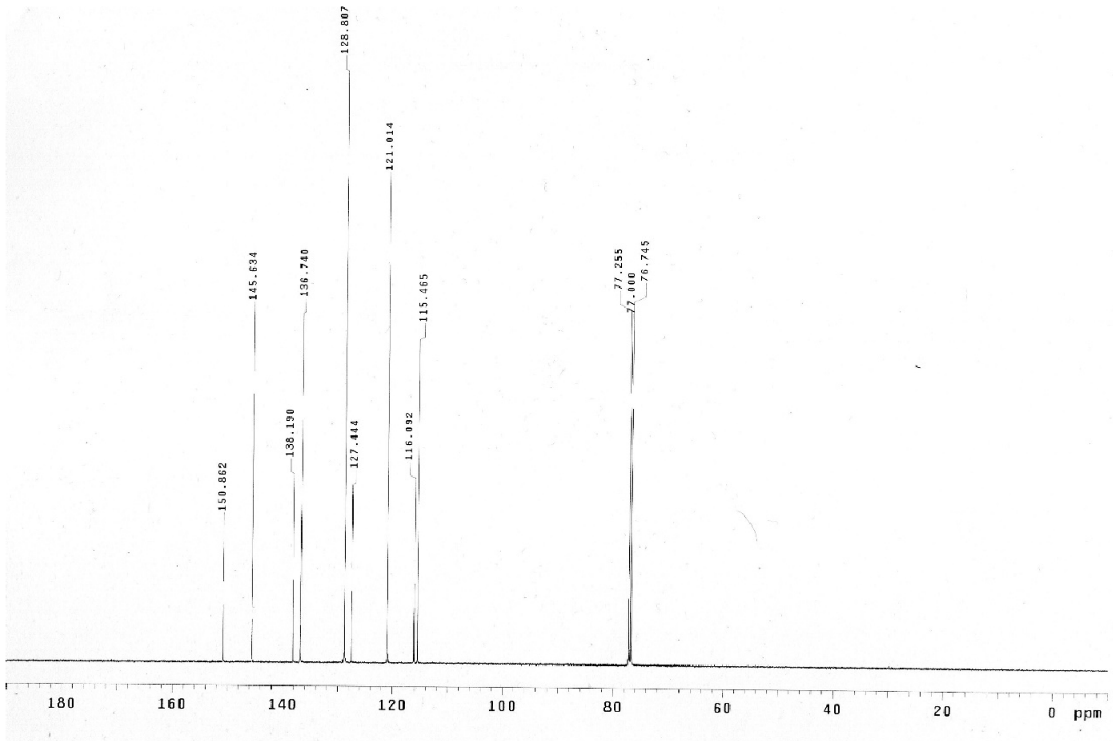

Figure S44: ^{13}C NMR of **19**

3-Chloro-N-(4-nitrophenyl)pyridin-2-amine (20)

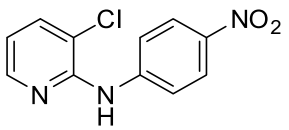

IR (KBr) ν 3359 cm^{-1}. ^{1}H NMR (400 MHz, $CDCl_3$) δ 6.88 (dd, J = 4.8, 8.0 Hz, 1H), 7.40 (br s, 1H, NH), 7.67 (dd, J = 1.6, 8.0 Hz, 1H), 7.86 (d, J = 8.8 Hz, 2H), 8.22 (d, J = 8.8 Hz, 2H), 8.23 (dd, J = 1.6, 4.8 Hz, 1H). ^{13}C NMR (100 MHz, $CDCl_3$) δ 29.7, 117.1, 117.3, 117.8, 125.3, 137.3, 145.7, 145.8, 149.8. EI-MS m/z: 251 (M+2), 249 (M+), 218, 202, 191, 168, 155, 140, 114, 102, 88, 76, 63, 50. EI-HRMS (m/z) calcd for $C_{11}H_8ClN_3O_2$: 249.0305; found: 249.0302.

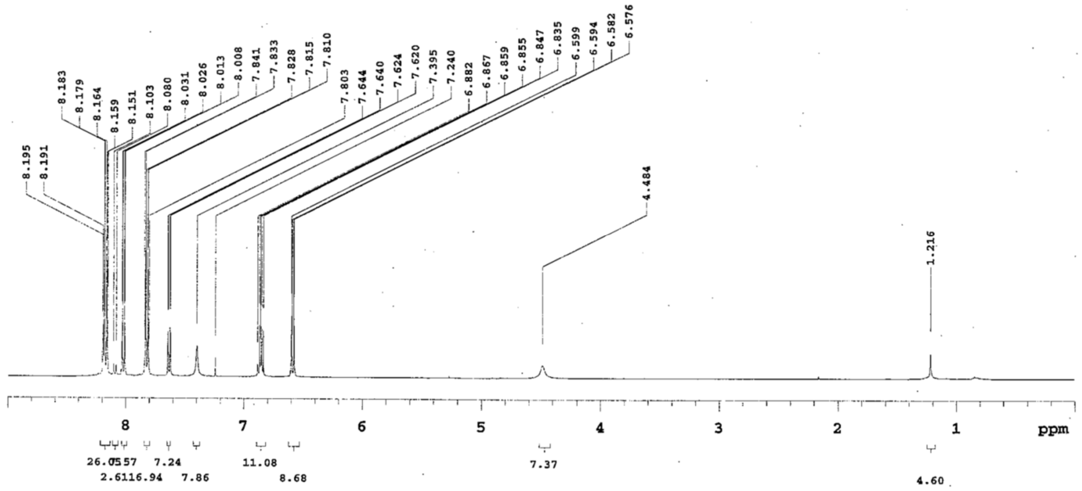

Figure S45: ^{1}H NMR of **20**

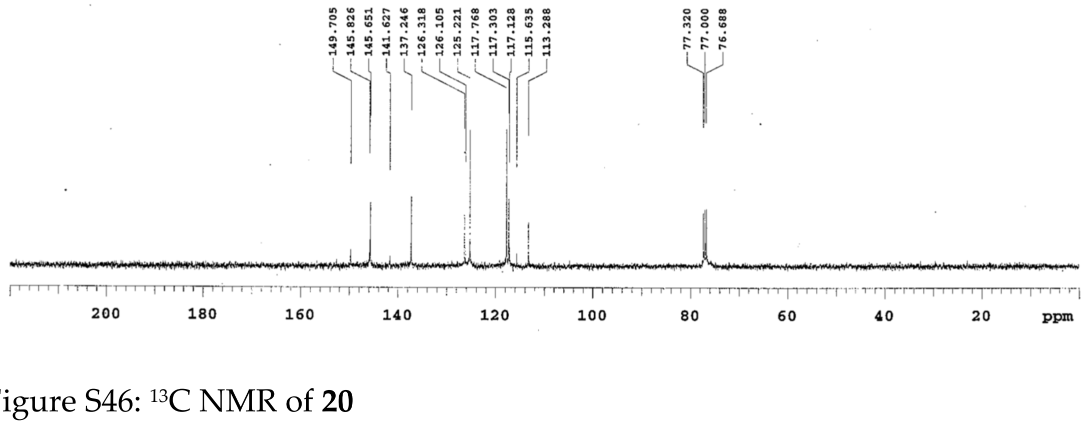

Figure S46: ^{13}C NMR of **20**
